# Supported employment interventions with people who have severe mental illness: Systematic mixed-methods umbrella review

**DOI:** 10.1371/journal.pone.0304527

**Published:** 2024-06-05

**Authors:** Emi Patmisari, Yunong Huang, Mark Orr, Sumathi Govindasamy, Emily Hielscher, Helen McLaren

**Affiliations:** 1 Institute for Mental Health and Wellbeing, College of Education, Psychology and Social Work, Flinders University, Bedford Park, South Australia, Australia; 2 Flourish Australia, Sydney Olympic Park, New South Wales, Australia; 3 QIMR Berghofer Medical Research Institute, Herston, Queensland, Australia; 4 School of Public Health, Faculty of Medicine, The University of Queensland, Brisbane, Queensland, Australia; Universidad Internacional de La Rioja, SPAIN

## Abstract

**Background/Aims:**

Primary and review studies show that supported employment interventions showed promise in assisting people with severe mental illness (SMI) in achieving successful employment and health-related outcomes. This umbrella review synthesises evidence from across review studies on supported employment interventions for individuals with SMI, to identify key findings and implementation challenges in relation to five key outcomes: (1) employment, (2) quality of life, (3) social functioning, (4) clinical/service utilisation, and (5) economic outcomes.

**Methods:**

A systematic search of eleven databases and registers (CINAHL, Cochrane, EmCare, JBI EBP, ProQuest, PsycINFO, PubMed, Scopus, and Web of Science, and Prospero and Campbell) was conducted to identify meta-analyses and systematic reviews on supported employment interventions for individuals with SMI, peer reviewed and published in English. Quality assessment and data extraction were performed using standardised Joanna Briggs Institute (JBI) tools. A mixed-methods synthesis approach was employed to integrate both quantitative and qualitative evidence.

**Results:**

The synthesis of 26 review studies primarily focused on the Individual Placement and Support (IPS) model among various supported employment interventions. Overall, combining supported employment with targeted interventions such as neurocognitive therapy and job-related social skill training showed a positive effect on employment (including job retention) and non-employment outcomes (e.g., health, quality of life, social functioning) relative to standard forms of supported employment for people with SMI. Contextual factors (intervention fidelity, settings, systemic barriers) were important considerations for intervention implementation and effectiveness.

**Discussion:**

Significant overlap of primary studies across 26 review studies exposed considerable variations in interpretation and conclusions drawn by authors, raising questions about their reliability. High volume of overlap reporting from the USA on IPS interventions in review studies is likely to have biased perceptions of effectiveness. There is no one-size-fits-all solution for supporting individuals with SMI in obtaining and maintaining employment. Tailoring strategies based on individual needs and circumstances appears crucial to address the complexity of mental health recovery. We propose creating centralised registries or databases to monitor primary studies included in reviews, thus avoiding redundancy.

**Other:**

This umbrella study was registered with PROSPERO (No. CRD42023431191).

## Introduction

Severe mental illness (SMI) is associated with some of the highest rates of unemployment and, when people with SMI are employed, most experience poor working conditions and high rates of discrimination [1]. Authors suggest unemployment rates among individuals with SMI may be up to 2.5 times higher than the unemployment rates of individuals with physical disabilities [2, 3]. In contrast, studies internationally show that the vast majority of people with SMI express a strong desire for work, assistance with getting a job, and support to sustain their workforce participation [e.g., 4–6]. Persistent high unemployment rates underscore the complex and multifaceted nature of the challenges faced by individuals with SMI in their quest for safe, meaningful, and sustained employment, as well as the need for ongoing research, policy development, and program refinement to address these barriers.

Access to employment offers critical benefits for individuals with SMI. Employment contributes to financial stability, social inclusion and community integration, boosting self-confidence and self-worth, reducing psychotic symptoms, minimising relapses, and contributing to a sense of personal existence and achievement [7–9]. Individuals with SMI frequently face negative reactions and are often denied equal opportunities and accommodations for employment. Challenges in achieving and maintaining employment for individuals with SMI span from individual to systemic barriers [10, 11]. However, denying employment to people with SMI not only exacerbates these challenges but also represents a denial of opportunities for occupation, active citizenship, and human rights, further marginalising this population and undermining their potential to contribute meaningfully to society [12–14]. Supported employment mechanisms are intended to enable individuals with SMI to participate in the workforce, safely and meaningfully. Supported employment programs are, therefore, important as they can significantly contribute towards these outcomes, enhance mental wellbeing, and mental health recovery [15, 16].

Whitley and Drake [17] emphasised the pivotal role of employment within the context of mental health recovery. Their model encompasses five fundamental domains: clinical, physical, functional, existential, and social. The functional domain, specifically employment, offers avenues for skill enhancement, interpersonal interactions, and engagement within the broader community. Each of these are crucial aspects in the recovery process as they contribute to an individual’s overall well-being [18]. Conversely, unemployment, job loss, and poor working conditions, can lead to negative emotions, including hopelessness and poor self-esteem, which can harm mental health and self-efficacy [19, 20]. In response, various supported employment models have been experimented worldwide with a view to addressing the unique needs and challenges faced by individuals with SMI in obtaining and maintaining employment. For the past two decades, effectiveness of various supported employment models has been assessed by a range of study authors across the globe, with varying outcomes.

Supported employment interventions refer to a wide array of services to help individuals with SMI secure and maintain employment in community settings. It employs strategies such as prevocational training, rapid job placement, transitional employment, on-the-job training, coordinated health services, and customised job development. Central to supported employment is the provision of both initial support and ongoing assistance, tailored to the specific requirements of each participant. This ensures a holistic method to vocational rehabilitation and integration, aiming to facilitate not just employment but also broader social and economic inclusion. Literature consistently highlights the positive effects of supported employment, having emerged when traditional psychiatric rehabilitation showed limited effectiveness in helping people with SMI to achieve satisfying lives [21–23]. Supported employment models originally developed for individuals with learning disabilities [24]. They have since been adapted for individuals with SMI, with a core emphasis on open competitive employment, e.g., provision of support to secure a job and maintain employment in the open job market concurrent with mental health or other treatments [25–27]. Individual Placement and Support (IPS), first developed and tested in US populations, is one of the most commonly known models of supported employment for SMI, incorporating rapid job search, tailored support services, and the integration of mental health and employment services [28, 29]. IPS appears to be the most extensively studied model [30], exhibiting notable enhancements in work-related achievements, quality of life, clinical outcomes, and demonstrated cost-effectiveness [31–33].

The next most known supported employment model is The Clubhouse, first established in the late 1940s [34, 35]. This model works on leveraging individual strengths in mental illness recovery, accentuate participatory, meaningful work, in a community-oriented environment. The principal aim is to promote social inclusion through work-related opportunities rather than strictly focusing on competitive employment. As a hybrid model, it combines elements from various other supported employment approaches and adapts these to meet the unique needs and circumstances of individuals with SMI [36, 37]. IPS and The Clubhouse share objectives of enhancing employability and global functioning for individuals with SMIs, however each model varies in its core methodologies, targeted outcomes, and evidence base. Despite the significant body of research documenting the effectiveness of supported employment interventions in enhancing employment opportunities for individuals with SMI [38, 39], unemployment rates among people with SMI remain high across the globe [1, 4, 5].

The quest for comprehensive understanding of supported employment in SMI populations is reflected in the increasing number of review studies. This expansive and varied body of research, encompassing both original investigations and subsequent reviews, has resulted in a range of conclusions. Bond et al. [40] attribute the inconsistencies to factors such as differences in model fidelity, non-integration of findings, over-emphasis on certain research methodologies, and inadequate consideration of contextual factors. For decades, vocational intervention studies for severe mental illness have encountered ongoing issues such as limited synthesis of a wide range of employment-related outcomes, constrained generalisability due to small trials of varying quality, and ambiguous terminology [41, 42]. Synthesis of the diverse body of evidence is needed, to identify the most effective components and factors influencing supported employment implementation and outcomes for people with SMI. This umbrella review offers a consolidated understanding of the overall evidence, identifying consistencies and discrepancies, from which to establish more robust conclusions. It aims to overcome potential limitations or biases in individual reviews and provides a broader perspective on the effectiveness, mechanisms, and contextual factors of supported employment interventions for individuals with SMI.

This umbrella review was guided by the question: ‘How do supported employment interventions impact individuals with severe mental illness, and what are the diverse factors influencing their outcomes?’ This was a mixed-methods synthesis review which explored the effectiveness of these interventions and the intricate interplay of service and program design, participant characteristics, contextual elements, and the perspectives of stakeholders.

## Methods

An umbrella review offers a valuable opportunity to address this knowledge gap by systematically synthesising findings from various reviews, including systematic reviews, meta-analyses, and scoping reviews [43]. This approach allows for a comprehensive overview of the existing evidence, identification of consistent findings across diverse studies, and recognition of areas that require further investigation [44, 45]. The protocol for the current study followed the Joanna Briggs Institute (JBI) guidelines for umbrella reviews [43]. The review protocol was registered on PROSPERO international prospective register of systematic reviews (No. CRD42023431191), and reporting is in accordance with the Preferred Reporting Items for Overviews of Reviews (PRIOR, see S1 Checklist) [46].

### Search strategy

Nine electronic journal databases were systematically searched to identify items reporting on review studies: CINAHL, Cochrane database of systematic reviews, EmCare (via Ovid SP), JBI database of systematic reviews and implementation reports, ProQuest (Social Sciences and Health & Medicine collections), PsycINFO (via Ovid SP), PubMed, Scopus, and Web of Science Core Collection (via ISI Web of Science). A comprehensive keyword search strategy was developed and piloted with the CINAHL database. This strategy facilitated the identification of primary keywords and language variations essential for the systematic exploration.

The principal search terms, ‘severe mental illness,’ ‘supported employment,’ and ‘review,’ were employed in combination. The search strategies were tailored and refined based on the specific requirements of each database (see S1 File). The search of each database was conducted on 6 July 2023. Review studies were limited to English language publications. No publication year or country restrictions were applied. Unpublished reviews were not sought. The reviews included in the analysis comprised review studies that themselves reviewed primary research published in peer-reviewed journals or other reputable sources, such as government reports, academic theses, and publications from respected research organisations. The first 10 pages of the Google Scholar search engine were searched on 15 August 2023. An examination of the references and citations in the identified review studies were undertaken, encompassing both backward and forward citation searching, also conducted on 15 August 2023. An updated hand search was conducted via Google Scholar on 11 April 2024, to seek out the most recent literature, however, no additional review studies were identified that fit the stringent inclusion criteria established for our umbrella review. Inclusion and exclusion criteria are outlined in Table 1 below.

**Table 1:** Study criteria.

Inclusion Criteria	Exclusion Criteria
• Reviews that evaluate the effectiveness of employment support interventions, programs, and strategies for individuals with severe mental illness. • Reviews that include studies published in peer-reviewed journals or other credible sources. • Reviews that focus legal working-age populations, according to country legislation in which this may differ. • Reviews that are published in English language.	**•** Reviews that do not focus on supported employment interventions for people who have severe mental illness. **•** Vocational or job training if not reporting on one or more outcomes of interest. **•** Reviews that do not provide clear information on the population, intervention/interest, comparison/context, and/or outcome of the included studies. **•** Reviews that are published in languages other than English. **•** Reviews that focus on under-age working populations (i.e., child labour).

### Population

Adults and adolescents who were recipients of supported employment interventions for people with SMI. SMIs are typically long-term mental illnesses involving substantial functioning impairment over multiple symptom and life domains. Disorders that are commonly considered an SMI include schizophrenia and other psychotic disorders, bipolar disorder, post-traumatic stress disorder, major depression, and eating disorders. No age parameter was applied to searching, however during screening we included populations of legal working age which differs across countries.

#### Interventions/Phenomena of interest

Strategies, techniques, and involvements applied in supported employment programs, inclusive of standard care, vocational rehabilitation and training, mental health, and adjunct treatments delivered concurrent with face-to-face and online or digital employment services and supports.

#### Comparator

Levels, duration, and types of individualised job development activities, job site supports and training, and ongoing supports. Some reviews synthesised employment intervention vs. a non-exposed control group, pre vs. post, user vs. non-user, whereas other reviews did not include any comparison (such as qualitative reviews).

#### Outcomes

Employment, quality of life, social functioning, mental health service utilisation, and economic outcomes. The reviews investigated the comprehensive effects of supported employment interventions on individuals with SMI, covering aspects such as job attainment, retention, satisfaction, improvements in quality of life and well-being, advancements in social integration and the capacity for meaningful role engagement, changes in hospitalisations and outpatient services usage, and variations in earnings alongside potential reductions in societal costs associated with unemployment and mental health challenges.

#### Types of review studies

Any type of review, including custom reviews, meta-analyses, meta-syntheses, narrative reviews, scoping reviews, realistic reviews, mixed methods reviews, qualitative evidence syntheses, and rapid reviews. Reviews could have included any kind of empirical primary studies: experimental, quasi-experimental, observational, mixed, and qualitative designs.

### Review selection, extraction and synthesis

A JBI data extraction form specific to umbrella reviews in JBI SUMARI (System for the Unified Management of the Assessment and Review of Information) was generated [47]. The specific information to be extracted was based on the research question and the inclusion/exclusion criteria (Table 1). Prior to review selection, the lead reviewer (EP) imported all citations into EndNote 20 and duplicates were removed. The resultant dataset was then exported into JBI SUMARI for screening and further analysis. All potentially relevant items were subjected to independent title and abstract screening, followed by full text screening, by two of the three reviewers (EP, HM & YH). Discrepancies in study inclusion were resolved by discussion (EP & HM).

Only reviews passing full-text screening were appraised. Two reviewers (EP & YH) used JBI critical appraisal checklists for Systematic Review and Research Syntheses (version 29 August 2017) independently within JBI SUMARI, with discrepancies resolved by a third reviewer (HM). Reviews were scored as ’good’ if over 80% of the appraisal attributes were affirmed, ’moderate’ if 50–80% of the attributes were affirmed, and ’poor’ if fewer than 50% were affirmed [48]. Evaluating review studies with JBI critical appraisal checklists ensures methodological rigor, yet a ’poor’ quality rating does not negate a study’s legitimacy or its potential contributions. Such ratings reflect specific design and reporting aspects, not the value of insights offered. Therefore, ‘poor’ quality review studies were included for a more balanced and unbiased view of the research landscape, and since these review studies may contain some valuable insights, evidence, or unique perspectives that contribute to the overall understanding of the topic.

Relevant data from eligible review studies were extracted, encompassing study details, participant characteristics, intervention specifics, outcome measures, results, and conclusions. After organising data and assessing study quality, the data were analysed. Subsequently, findings were interpreted, considering research questions and data trends, strengths of evidence, and implications. The results were synthesised narratively, adhering to the guidelines established by the Joanna Briggs Institute.

Overlapping primary studies among review studies can present significant methodological challenges. When conducting an umbrella review, overlap may misdirect findings due to their significant influence on both quantitative and qualitative analyses. In our evaluation of the extent of overlap, we employed the Corrected Covered Area (CCA) method, developed by Pieper et al. [49], using the formula CCA = (N–r)/(rc–r). Within this equation, ’N’ denotes the aggregate number of incorporated publications (including those enumerated more than once), ’r’ symbolises the quantity of unique publications, and ’c’ encapsulates the total number of reviews. In addition, we utilised the Graphical Representation of Overlap for OVErviews (GROOVE), which further aids in assessing overlap [50]. This tool provides the number of primary studies and reviews included in the matrix, the absolute number of overlapped, and non-overlapped primary studies, and an overall CCA assessment. GROOVE also detailed CCA analysis for each possible pair of reviews (or "nodes"), structural missingness in the matrix. Structural missingness (“X”) refers to a specific type of missing data in research, for instance if a review was published in 2020 and it claims to include primary studies published up to that point, it cannot include a primary study published in 2023. In this case the formula of CCA is (N–r)/(rc–r–X). To delineate the magnitude of overlap, we utilised pre-established thresholds: Less than 5% is indicative of slight overlap, 5 to <10% is categorised as moderate, 10 to <15% is characterised as high, and any rate exceeding 15% is classified as exceedingly very high overlap [50].

Data synthesis using a mixed-method overview of reviews involved systematically aggregating and analysing findings from the multiple qualitative and quantitative review studies included. We summarised and narratively synthesised quantitative data from the included review studies, rather than performing meta-analysis, as this approach avoids the pitfalls of combining diverse quantitative data, such as heterogeneity in study designs and populations. When presenting the results, we organised the interventions into four distinct categories, Standard Supported Employment, Augmented Supported Employment, Vocational Rehabilitation and Training, and Standard Care. Additionally, we created a matrix to classify the findings based on five critical outcomes: employment, quality of life, social functioning, clinical/service use, and economic outcomes.

## Results

### Selection of review studies

**Fig 1:**
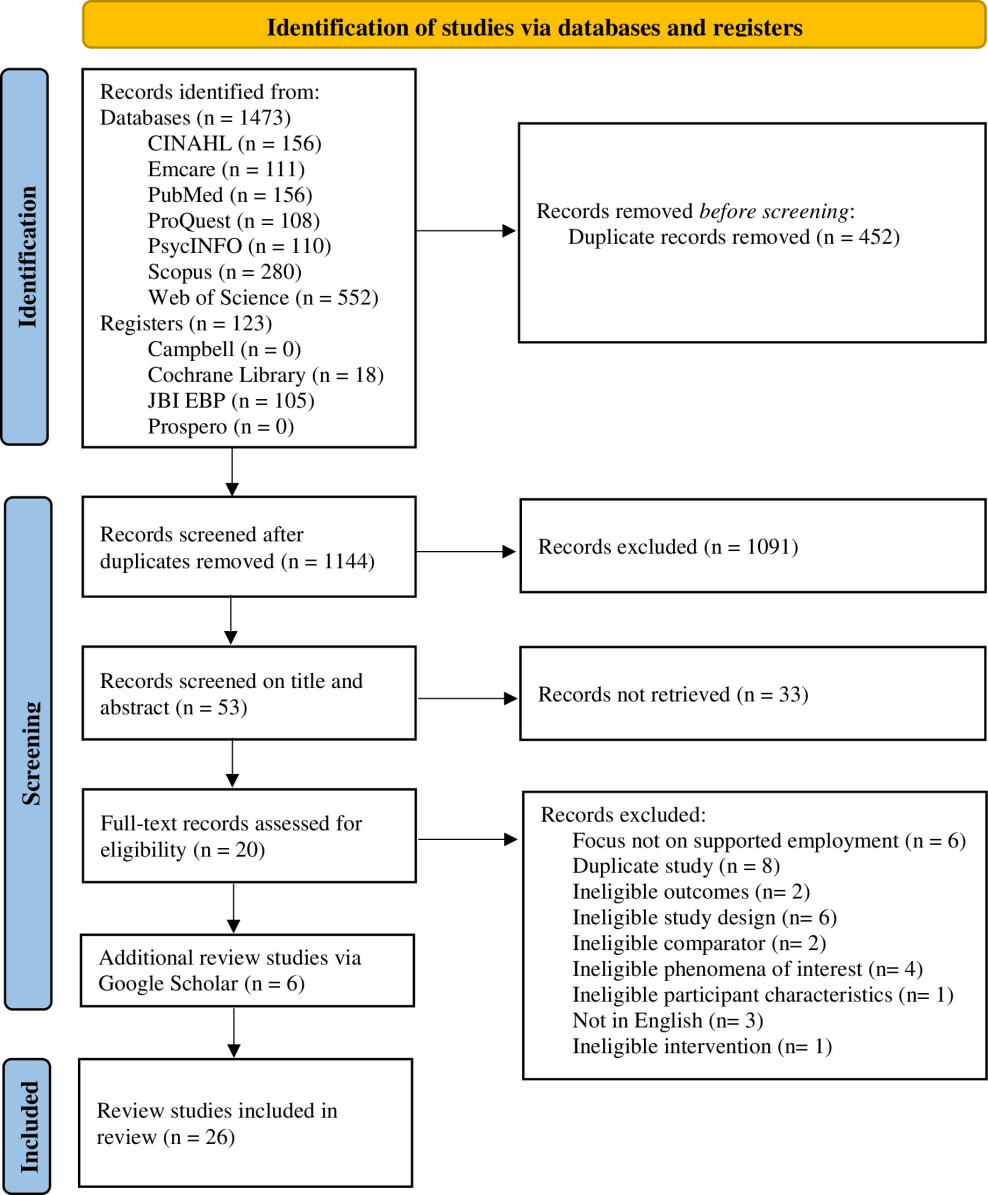
PRISMA flow diagram for included studies.

Results from the systematic search revealed a total of 1473 records retrieved from various databases. These databases included CINAHL with 156 records, Emcare with 111 records, PubMed with 156 records, ProQuest with 108 records, PsycINFO with 110 records, Scopus with 280 records, and Web of Science with 552 records. A total of 123 records were identified from registers, 18 from Cochrane and 105 records from JBI, while Campbell and Prospero had no records associated with the search. Relevant titles and abstracts were assessed for eligibility (*n* = 53), resulting in 20 reviews that met the inclusion criteria. Six additional reviews sourced through hand searching were included, resulting in a total of 26 review studies included. See Fig 1, PRISMA [51].

### Review characteristics

The 26 reviews varied by review design, participant characteristics, interventions, and outcomes. They collectively included reviews of 497 primary studies. Out of 26 reviews, 16 did not specify a country or indicate a multi-country focus. Eight reviews encompassed studies from multiple countries, and one review was exclusively focused on primary studies conducted in Australia [52] and another included only UK primary studies [53]. Participant counts in each review varied from 258 to 10,825. A total of 73,304 participants were included in 24 of the reviews (aggregate number of participants includes overlap in primary studies). The two other reviews provided a participant range of 14 to 2096 [52] and 37 to 147 [54]. The included reviews encompassed various types, including systematic reviews (*n* = 11), Cochrane reviews (*n* = 3), meta-analyses (*n* = 7), meta-ethnographic reviews (*n* = 2), a scoping review (*n* = 1), a scoping and systematic review (*n* = 1), and an integrative review (*n* = 1). Reviews were published between 2001 and 2023, with the primary studies included ranging from 1963 to 2021. Most reviews (85%) primarily examined vocational outcomes in evaluating the effectiveness of diverse employment programs, while only a small number (*n* = 4) delved into non-vocational aspects, such as quality of life, social functioning, clinical, or economic outcomes, and experiences. Of the 26 review studies, 25 focused on either the effectiveness of IPS alone or the comparison of effectiveness of IPS with other alternative interventions or services as usual, and one on the Clubhouses alone [55].

In terms of quality assessment, several reviews (*n* = 7) were rated as good, indicating a high level of methodological rigor and quality in their respective research syntheses. A substantial number of reviews (*n* = 7) received poor ratings, with scores falling below 50%. These reviews exhibited shortcomings in their methodological approaches and reporting. The majority of the reviews, however, fell into the moderate category (*n* = 12), suggesting a middle ground in terms of quality and reliability (Table 2).

**Table 2:** Critical appraisal of included systematic reviews and research syntheses.

	Reviews	Q1	Q2	Q3	Q4	Q5	Q6	Q7	Q8	Q9	Q10	Q11		
1	Abidin et al., 2021	U	Y	Y	Y	Y	Y	U	N	N	Y	Y	64%	Moderate
2	Aguey-Zinsou et al., 2022	Y	Y	Y	Y	Y	Y	Y	N	N	Y	Y	82%	Good
3	Bond, Drake & Becker, 2008	U	Y	U	U	N	N	U	N	N	Y	Y	27%	Poor
4	Bond et al., 2012	U	Y	U	U	Y	U	Y	Y	N	Y	Y	55%	Moderate
5	Bond et al., 2023	U	Y	Y	Y	U	Y	Y	Y	N	Y	Y	73%	Moderate
6	Campbell et al., 2011	Y	Y	U	U	U	U	U	Y	N	Y	Y	45%	Poor
7	Carmona et al., 2017	U	Y	Y	Y	Y	Y	Y	Y	Y	U	Y	82%	Good
8	Charette-Dussault & Corbiere, 2019	U	Y	U	Y	Y	Y	U	U	N	Y	U	45%	Poor
9	Charzyńska et al., 2015	U	Y	Y	U	N	N	N	N	N	Y	U	27%	Poor
10	Chen & Lal, 2020	Y	Y	Y	U	NA	NA	Y	NA	NA	Y	Y	86%	Good
11	Crowther et al., 2001a	U	Y	Y	Y	U	Y	U	Y	N	Y	N	55%	Moderate
12	Crowther et al., 2001b	Y	Y	Y	Y	Y	Y	Y	Y	Y	Y	Y	100%	Good
13	Dewa et al., 2018	Y	Y	U	Y	Y	Y	Y	U	U	Y	Y	73%	Moderate
14	Frederick & VanderWeele, 2019	U	Y	U	U	U	U	U	Y	N	N	U	18%	Poor
15	Haffernan & Pilkington, 2011	Y	Y	Y	U	Y	Y	N	N	N	Y	Y	64%	Moderate
16	Johanson et al., 2023	Y	Y	Y	U	Y	Y	U	U	N	U	Y	55%	Moderate
17	Kinn et al., 2021	U	Y	U	Y	Y	U	U	Y	U	Y	Y	55%	Moderate
18	Kinoshita et al., 2013	U	Y	Y	Y	Y	Y	Y	U	Y	Y	Y	82%	Good
19	Mallick & Islam, 2022	U	Y	Y	U	Y	U	U	N	N	Y	Y	45%	Poor
20	McKay et al., 2018	U	Y	Y	Y	Y	Y	U	U	N	Y	Y	64%	Moderate
21	Metcalfe et al., 2018	U	Y	Y	Y	U	U	Y	Y	N	Y	N	55%	Moderate
22	Modini et al., 2016	U	Y	Y	Y	Y	Y	Y	Y	Y	Y	N	82%	Good
23	Moen et al., 2020	U	Y	U	Y	Y	U	U	U	N	Y	Y	45%	Poor
24	Suijkerbuijk et al., 2017	U	Y	Y	Y	Y	Y	Y	Y	Y	U	Y	82%	Good
25	Twamley et al., 2003	U	Y	Y	Y	U	U	U	Y	U	Y	Y	55%	Moderate
26	Wallstroem et al., 2021	U	Y	Y	Y	U	U	Y	Y	N	U	Y	55%	Moderate

Y = Yes; N = No; U = Unclear; NA = Not applicable; Good: >80% Moderate: 50–80% Poor: <50%

Across the 26 reviews, a total of 497 reported primary studies were identified and included as part of the analysis. Among the 26 reviews, only 2 had no overlap of primary studies included with other reviews: Johanson et al. [56] which focused on cost-effectiveness, and Mallick and Islam [52] which examined employment in the Australian context. Taking into account study overlap, the remaining 24 reviews included 291 discrete primary studies. After adjusting for the structural zeros, the corrected covered area was 14.69%. This adjusted metric provides a more refined assessment of the covered area, considering both overlap and structural zeros (Table 3). These findings highlight a more accurate representation of the coverage of primary studies by the reviews, aiding in drawing conclusions from the collected data.

**Table 3:** Overlapping primary studies corrected covered area results.

Overall results		
Number of reviews		26
Number of primary studies reported in 26 reviews		497
Number of reviews without overlap		2
Number of columns (number of reviews with overlap)	c	24
Number of rows (number of index publications)	r	78
Number of included primary studies (discrete studies)	N	291
Covered area	N/(rc)	15.54%
Corrected covered area	(N-r)/(rc-r)	11.87%
Interpretation of overlap	**High overlap**	
Structural zeros	X	344
Corrected covered area (adjusting by structural zeros)	(N-r)/(rc-r-X)	14.69%

**Fig 2:**
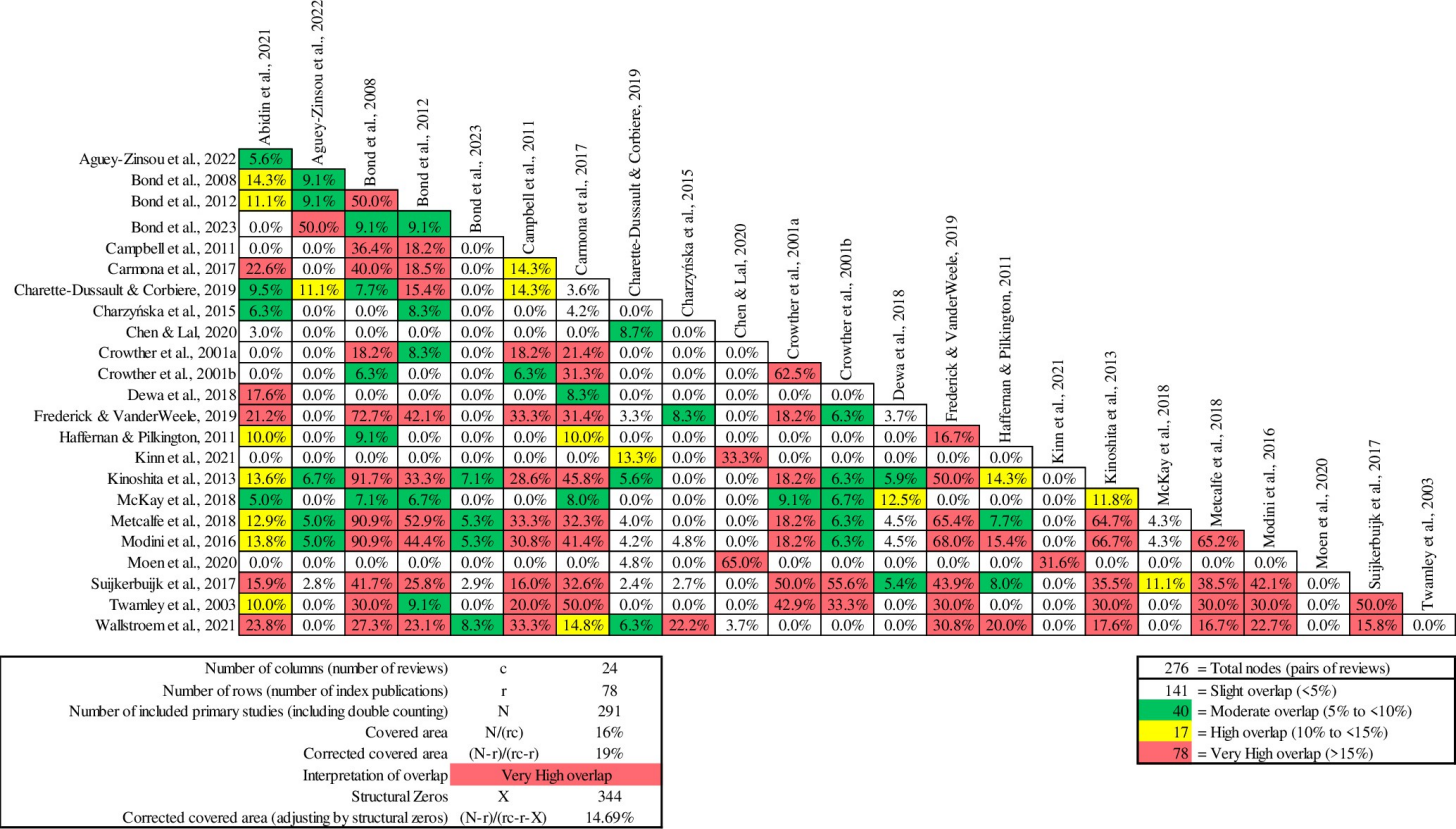
Graphical Representation of Overlap for Overviews (GROOVE) results.

Fig 2 presents the GROOVE results, highlighting the degree of overlap between pairs of reviews. Among the 276 pairs of reviews examined, 141 pairs reflect minimal overlap, 40 pairs show moderate overlap, 17 pairs encounter a higher degree of shared content, and 78 pairs represents a substantial overlap, with more than 15% of primary studies replicated between reviews within each pair. Bond et al. [40] exhibited the most substantial degree of overlap, aligning with 11 other reviews, reaching a significant 91.7%. This suggests a notable amount of redundancy or similarity between Bond et al., 2008, and these 11 other reviews in terms of the research they included or discussed.

### Intervention typology

Within the scope of the included reviews in the current umbrella study, terminology used to describe interventions varied with many terms used interchangeably. Terminological diversity may be attributed to the nuanced ways in which various supported employment programs are implemented and adapted to suit specific populations or clinical contexts. Utilising findings from the 26 review studies, we classify these interventions into four distinct intervention typologies as was described across the reviews, e.g., Standard Supported Employment, Augmented Supported Employment, Vocational Rehabilitation and Training, and Standard Care, identifying in Table 4 the five dominant intervention outcomes from across the review studies, e.g., employment, quality of live, social functioning, clinical/service use, and economic. This provided a structured framework for comprehending and synthesising the multifaceted strategies employed within the context of overall supported employment interventions. Vocational Rehabilitation and Training is encapsulated within our conceptual definition of supported employment interventions since some of the reviews variously included this in their definitions and scope. This inclusion recognises the integral role that vocational training and rehabilitation services play in preparing individuals for the workforce, enhancing their employability, or supporting their performance whilst in employment settings. Interventions categorised under Standard Care were examined within this review, when reported for purposes of contextualising the effectiveness of employment interventions, or comparative analyses of innovations, against standard or pre-existing models which were not clearly reported. Inclusion of four typologies allows for a comprehensive synthesis of the landscape in which supported employment operates, acknowledging that a range of intervention strategies are implemented to support employment outcomes for individuals.

**Table 4:** Alignment of included reviews with intervention typologies and outcome framework.

Author(s), year	Intervention typologies	Employment	Quality of Life	Social Functioning	Clinical/ Service Use	Economic
Abidin et al., 2021	Standard Supported Employment	**✓**	**✓**	**✓**	**✓**	**✓**
	Augmented Supported Employment	**✓**	**✓**	**✓**	**✓**	
	Vocational Rehabilitation and Training	**✓**			**✓**	
Aguey-Zinsou et al., 2022	Standard Supported Employment	**✓**				
	Augmented Supported Employment	**✓**				
	Vocational Rehabilitation and Training	**✓**				
	Standard Care	**✓**				
Bond et al., 2008	Standard Supported Employment	**✓**				
Bond et al., 2012	Standard Supported Employment	**✓**		**✓**	**✓**	**✓**
Bond et al., 2023	Standard Supported Employment	**✓**				
Campbell et al., 2011	Standard Supported Employment	**✓**				
Carmona et al., 2017	Standard Supported Employment	**✓**				**✓**
	Augmented Supported Employment	**✓**				
	Vocational Rehabilitation and Training	**✓**				
	Standard Care	**✓**				
Charette-Dussault & Corbiere, 2019	Standard Supported Employment	**✓**				
	Vocational Rehabilitation and Training	**✓**				
	Standard Care	**✓**				
Charzyńska et al., 2015	Standard Supported Employment		**✓**	**✓**	**✓**	
Chen & Lal, 2020	Standard Supported Employment	This qualitative review focuses on stakeholders’ experiences and perspectives, emphasising the need for improving collaboration between mental health and vocational teams.				
Crowther et al., 2001a	Standard Supported Employment	**✓**	**✓**		**✓**	**✓**
	Augmented Supported Employment	**✓**			**✓**	**✓**
	Vocational Rehabilitation and Training	**✓**			**✓**	**✓**
Crowther et al., 2001b	Standard Supported Employment	**✓**	**✓**	**✓**	**✓**	
	Vocational Rehabilitation and Training				**✓**	**✓**
Dewa et al., 2018	Standard Supported Employment					
	Augmented Supported Employment	**✓**				**✓**
Frederick & VanderWeele, 2019	Standard Supported Employment	✓	**✓**		**✓**	**✓**
Heffernan & Pilkington, 2011	Standard Supported Employment	**✓**				
Johanson et al., 2023	Standard Supported Employment	**✓**				**✓**
	Augmented Supported Employment	**✓**			**✓**	**✓**
	Vocational Rehabilitation and Training	**✓**			**✓**	**✓**
Kinn et al., 2021	Standard Supported Employment	This qualitative review emphasises the Employment Specialists’ contributions in creating personalised job support strategies and the importance of their relationship with clients.				
Kinoshita et al., 2013	Standard Supported Employment	**✓**	**✓**	**✓**	**✓**	**✓**
Mallick & Islam, 2022	Standard Supported Employment	**✓**				
McKay et al., 2018	Standard Supported Employment	**✓**	**✓**	**✓**	**✓**	
Metcalfe et al., 2018	Standard Supported Employment	**✓**				
Modini et al., 2016	Standard Supported Employment	**✓**				
Moen et al., 2020	Standard Supported Employment	This qualitative review highlights the challenges and frustrations in cooperation between employment specialists, social workers, and mental health clinicians within the IPS framework.				
Suijkerbuijk et al., 2017	Standard Supported Employment	**✓**				
	Augmented Supported Employment	**✓**				
	Vocational Rehabilitation and Training	**✓**				
	Standard Care	**✓**				
Twamley et al., 2003	Standard Supported Employment	**✓**				
	Vocational Rehabilitation and Training	**✓**				
Wallstroem et al., 2021	Standard Supported Employment	**✓**	**✓**		**✓**	

#### Standard supported employment (*n =* 26 review studies)

Standard Supported Employment refers to a comprehensive employment model designed to assist individuals with significant barriers to employment, including mental illnesses and other disabilities, in gaining and maintaining competitive jobs. This model encompasses various sub-models such as the IPS model with its fidelity variations, vocational case management adopting IPS, accelerated transitional or supported employment program, family-aided assertive community treatment and vocational specialist, Clubhouse model, individual enabling support, IPS modified for anxiety and mood disorders, social enterprise, paid and job placement plus weekly support, Assertive Community Treatment plus vocational specialists, or Indianapolis vocational intervention program.

#### Augmented supported employment (*n =* 7 review studies)

This second typology is an enhanced form of supported employment, combined with one or more additional interventions that range from extra job coaching, and cognitive remediation to symptom management. Augments aim to provide a more integrated approach to employment support for individuals with severe mental illnesses. Seven review studies included primary studies relevant to this category in which Standard Supported employment was augmented with IPS plus social skills training, IPS plus internship program, supported employment plus supported education, individual enabling support, IPS modified for anxiety and mood disorders, neurocognitive enhancement therapy plus hybrid transitional and supported employment, IPS plus Assertive Community Treatment, IPS plus Cogrehab software (cognitive training), IPS plus thinking skills for work, IPS plus workplace fundamental skills module, IPS plus cognitive remediation and social skills, supported employment plus job-related or symptom-related skills training, and supported employment plus sheltered employment.

#### Vocational rehabilitation and training (*n =* 9 review studies)

Nine reviews included primary studies focused on Vocational Rehabilitation and Training. These encompassed a wide range of interventions, each tailored to the unique needs and circumstances of individuals in acquiring the skills, competencies, and support necessary to enter, maintain, or regain employment. Across primary studies included, these interventions included, but were not limited to: psychiatric vocational rehabilitation, cognitive intervention, virtual reality-based pre-vocational training, cognitive adaptation training, industrial therapy, job in jeopardy, paid and job placement, behavioural interventions plus paid and job placement, cognitive enhancement therapy, vocational integrated program of Assertive Community Treatment, Assertive Community Treatment plus family psychoeducation groups, prevocational training, prevocational training plus payment, prevocational training plus psychosocial interventions, accelerated entry to transitional employment, symptom-related skills training, incentive therapy, cognitive training or social skills training.

#### Standard care (*n =* 4 review studies)

Standard Care refers to a fundamental framework of mental health services and interventions that offer essential treatment, support, and rehabilitation to individuals with mental health conditions within their local communities. In the context of evaluating supported employment interventions, Standard Care served as a comparative baseline in four of the reviewed studies. This approach typically included a range of services such as Assertive Community Treatment, psychiatric care, medication management, or access to mental health professionals to address mental health needs. The inclusion of Standard Care in this review underscores the potential advantages of supported employment by directly contrasting it with the outcomes achieved through traditional mental health support services.

### Impact of various supported employment interventions

Among the reviewed studies (Table 5), twenty primarily focused on evaluating employment outcomes, including preparing for, seeking, obtaining, maintaining, and regaining employment, while six others explored various aspects related to: barriers experienced by people with SMI [57], individual and stakeholder experiences of IPS [58–60], cost-effectiveness [56], and recovery-related outcomes such as symptom remission, global functioning, and quality of life [61, 62].

**Table 5:** Overview of review characteristics.

	Author(s), year	Review aims	Participants (characteristic/total number)	Description of interventions/ phenomena of interest	Number, year range, and types of studies included	Outcomes assessed
1	Abidin et al., 2021	To assess the effectiveness of employment programs considering both vocational and non-vocational outcomes.	Adults aged 18–65 with schizophrenia and other SMI. 3165 participants, 2191 males, 974 females.	Supported employment, integrated supported employment, vocational rehabilitation, cognitive intervention, and virtual reality-based vocational training.	24 studies from 2000 to April 2020. The paper only included randomised controlled trials (RCTs) that examined the effectiveness of intervention programs for schizophrenia and other SMI.	Primary outcomes: Employment rate, job tenure, days/hours of working and job change.
						Secondary outcomes: Admission and re-admission rate, social functioning, quality of life, psychiatric symptoms, self-esteem and wellbeing.
2	Aguey-Zinsou et al., 2022	To identify and synthesise the available evidence on the employment processes and outcomes for young adults experiencing psychosis.	Young adults aged 14–30. 9262 participants from 29 intervention studies that are included in this review.	Cognitive Adaption Training, Community Treatment Orders, Early Intervention, Extended Early Intervention, Industrial therapy, IPS and IPS adapted to Include unpaid internships, Job in Jeopardy, Medication Discontinuation, Medication, Supported Education and Supported Employment program, Vocational Case Management adopting IPS principles.	29 intervention studies from 1973 to 2019. Cohort study (n = 20), quasi-experiment (n = 5), Cross sectional (n = 1), RCT (n = 3).	1. Employment Rates and Economic Costs of Unemployment
						2. Intervention Impact
						3. Employment Processes; preparing for, seeking, obtaining, keeping, and re-obtaining.
3	Bond et al., 2008	To assess competitive employment outcomes in randomised controlled trials (RCTs) that examine evidence-based supported employment for individuals dealing with severe mental illness.	534 IPS participants, 610 Control participants.	Individual Placement and Support (IPS) vs control (skills training non-integrated, sheltered workshop, psychosocial rehabilitation (PSR), brokered SE, diversified placement approach, stepwise conventional services, vocational rehabilitation, and TAU)	11 studies (7 US, 4 outside US) between 1996 and 2008. RCTs of IPS programs.	Employment rates, time to initial employment, weeks worked on an annual basis, and tenure in the longest-held job during the follow-up period
4	Bond et al., 2012	To analyse the effectiveness of Individual Placement and Support (IPS) for people with severe mental illness.	1063 participants (mean = 70.9 per study).	Individual Placement and Support (IPS)	15 studies (9 US, 6 Outside US) from 1996 to 2012. RCTs of IPS programs.	1. Competitive Employment Outcomes; Employment Rate, Days to First Job, Weeks Worked during Follow-up, and Hours Worked.
						2. Non-competitive Employment, Program Retention, and Nonvocational Outcomes.
5	Bond et al., 2023	To evaluate the effectiveness of Individual Placement and Support (IPS) interventions for young adults with serious mental illness.	357 IPS participants and 340 control participants. Young adults or transition-age youth with first-episode psychosis.	Individual Placement and Support (IPS)	7 studies included in meta-analysis from September 2019 to March 2022. 4 RCTs of IPS for young adults with early psychosis, and 3 RCTs of other young adult subgroups.	1. Competitive Employment Outcomes (also a comparison between effect on young adults and older adults), including job duration
						2. Education Outcomes
						3. Combined Employment and Education Rate
6	Campbell et al., 2011	To identify which sub-groups of clients with severe mental illness (SMI) benefited from evidence-based supported employment.	307 adults with SMI and 67 in comparison group (374 in total).	Individual Placement and Support (IPS)	4 studies included between 1991 and 2002. RCTs of IPS programs.	Competitive Employment Outcomes (obtaining a job, total weeks worked, and job tenure).
7	Carmona et al., 2017	To assess the efficacy of vocational interventions for individuals with schizophrenia spectrum disorder, and to identify predictor variables that may influence employment outcomes.	866 people with schizophrenia, 299 with schizoaffective disorder and 1199 with schizophrenia spectrum diagnosis (2364 in total).	Individual Placement and Support (IPS); Paid and job placement; Behavioural interventions, paid, and job placement; Neurocognitive enhancement therapy + hybrid transitional and supported employment; Accelerated transitional employment program; Accelerated condition of supported employment; Paid, job placement & weekly support group; Cognitive enhancement therapy; Assertive community treatment (ACT) + IPS; Supported employment; Indianapolis vocational intervention program, paid and job placement; ACT from supported employment; ACT and family psychoeducation groups; Family-aided ACT + vocational specialist; Psychosocial rehabilitation; Psychiatric vocational rehabilitation; Integrated supported employment.	25 studies with most studies were conducted in the United States (n = 19), China (n = 2), Canada (n = 1), Sweden (n = 1), and the UK (n = 1), European countries (n = 1). The search covered the period from 1986 to December 31, 2015. Studies are all RCTs.	Primary Employment Outcomes (both competitive and any other employment): job placement and job tenure.
						Secondary Outcomes: wages earned from competitive employment.
8	Charette-Dussault & Corbiere, 2019	To analyse and synthesise studies focusing on barriers/obstacles to employment for people with SMI.	4825 participants (varying from 56 to 2326) in 26 quantitative studies. 638 respondents in 9 qualitative studies (varying from 9 to 279 participants).	Supported employment programs (SEP), individual placement and support (IPS)	35 studies included from 1997 to 2017. 8 quantitative cross-sectional, 18 prospective studies, and 9 qualitative studies.	Barriers experienced by people with SMI in their path to employment.
9	Charzyńska et al., 2015	To explore the correlation between various forms of employment and specific non-vocational markers of recovery.	5600 participants	Supported employment, IPS, competitive employment	18 studies between 1993 and 2013. Cross-sectional; naturalistic longitudinal; clinical controlled trial; randomised controlled trial.	Symptom remission, cognitive function, social and emotional functioning, and quality of life.
10	Chen & Lal, 2020	To synthesise qualitative literature to understand how stakeholders, experience and perceive IPS and consider the implications for future occupational therapy practice and research.	733 participants (aged 18–65). Of the 19 studies that examined client perspectives, 9 recruited minorities and Caucasians, 9 did not report ethnicity, and 1 recruited only Caucasians.	Individual Placement and Support (IPS)	26 studies included from 1995 to 2018.	The perception of clients, employment specialists, and employers regarding features of IPS that clients and other stakeholders appreciate, factors hindering IPS, factors contributing to IPS success, rules and regulations of welfare system conflicting with IPS principles and impacts of IPS on mental health and well-being.
11	Crowther et al., 2001a	To determine the most effective way of helping people with severe mental illness to obtain competitive employment	Total 1951 participants. 1204 participants from 5 prevocational training with standard care trials. 256 participants in one supported employment with standard care trial. 491 participants in 5 supported employment with prevocational training trials. Aged 18 to 65 with SMI	Prevocational training, supported employment, and standard community care	11 RCT studies were included, published between 1994 and 2000.	Primary Outcomes: Number of subjects in competitive employment.
						Secondary Outcomes: Other employment outcomes, clinical outcomes, and costs.
12	Crowther et al., 2001b	To assess the effects of Pre-vocational Training and Supported Employment for people with SMI.	2539 participants, aged 18–65; and suffering from severe mental disorder defined as: schizophrenia and schizophrenia-like disorders; bipolar disorder; or depression with psychotic features.	Prevocational training, supported employment, enhanced approaches, and standard care.	18 RCTs were included published between 1963 and 1998	Primary Outcomes: Number in competitive employment.
						Secondary Outcomes: Other employment outcomes, clinical outcomes, and costs.
13	Dewa et al., 2018	To examine the effectiveness of augmented versus standard IPS for people with SMI.	People over 18 with SMI. A total of 929 participants from 5 studies were included	IPS program with an augmentation that was compared to standard IPS	Seven articles from 5 RCT studies were included published between 2002 and January 2016.	Employment outcome, which includes employment rate, job tenure, and wages/income.
14	Frederick & VanderWeele, 2019	To assess the effectiveness of IPS treatments on vocational and non-vocational outcomes	A total of 5664 participants (2852 undergoing IPS, and 2812 Treatment as Usual). Age not reported.	Supported employment: Individual Placement and Support (SE IPS)	30 RCT studies were included published between 1996 and 2017.	Vocational outcomes: Competitive employment), time to first competitive employment job, job tenure, total time worked, and income). Non-vocational outcomes (quality of life, global functioning, and mental health).
15	Heffernan & Pilkington, 2011	To examine the evidence of effectiveness of the IPS model in the UK.	1181 participants	Individual Placement and Support (IPS)	5 studies, 2004–2010. 2 RCTs, 1 Cohort study, 1 Naturalistic study, 1 small evaluation.	Employment rates and job tenure.
16	Johanson et al., 2023	To identify and summarise evidence of cost-effectiveness of Return-to-Work interventions for persons with mental health disorders.	A total of 2283 participants were recorded in 8 studies. The participants of one study was not reported. People aged 18–67 years with mental health disorders who were on sick leave, fully or partially employed or unemployed.	SE IPS, Employment Specialist Integrated in mental healthcare service, individual support according to the principles of IPS, IPSE (IPS + Cognitive remediation and social skills training), Community mental health service or early intervention teams, IPS MA added to Service as Usual, OT added to depression treatment as usual, 3 OT phases.	9 studies included, 2002–2021	Health-economic Outcomes (Cost-utility, cost-effectiveness, cost-minimisation, cost-benefit)
17	Kinn et al., 2021	To synthesise the perspectives of Supported Employment clients, employment specialists, and their supervisors in providing job support.	101 IPS clients living with SMI and 147 employment specialists (including 6 IPS supervisors).	Individual Placement and Support (IPS)	16 qualitative studies, between 2006 and 2018.	The perspectives of IPS/SE clients, employment specialists, and supervisors on job support.
18	Kinoshita et al., 2013	To review the effectiveness of supported employment.	People aged 16–70 with severe mental illness. A total of 2265 people within 14 studies.	Supported employment: IPS and Augmented Supported Employment; 2. Other Vocational Approaches; 3. Treatment as Usual	A total of 14 RCT studies were included, published between 1996 and 2010.	Days in competitive employment, long-term employment, education, leaving the study early, global state, mental state, service use, quality of life, social/general functioning, adverse effects, economic costs (excluding housing costs).
19	Mallick & Islam, 2022	To investigate the impact of IPS co-location partnerships between adult community mental health teams (ACMHTs) and disability employment services (DES) on employment outcomes and consumer choice of work for adults with SMI.	Adults (18–65) with SPMI (serious persistent mental illness), participants ranging from n = 14 to n = 2096	IPS, DES practice, funding, policy, and reform within the Australian mental health system.	12 studies were included, 01 January 2017–30 August 2021. 2 quantitative studies, 7 qualitative studies, 3 mixed-methods studies.	Barriers to IPS Implementation, employment versus unemployment, IPS and non-IPS co-location partnerships, DES within the Australian mental health system, and barriers to participation in DES programs.
20	McKay et al., 2018	To conduct a systematic review of articles providing a comprehensive understanding of what is known about the Clubhouse Model.	The ages of the participants were not reported. There was a total of 10825 participants.	The Clubhouse Model: Which includes employment at prevailing wages in the wider community through Transitional Employment (TE), Supported Employment (SE), and Independent Employment (IE).	52 RCT studies met the selection criteria, published between 1948 and 2015	Six outcome domains including: (1) employment including TE, SE, and IE, (2) hospitalization/recidivism, (3) quality of life/satisfaction, (4) social relationships, (5) education, and (6) health promotion activities.
21	Metcalfe et al., 2018	To assess the impact of site-level moderators on the likelihood that IPS recipients, compared with recipients of alternative vocational services, achieved competitive employment.	Adults with SMI (aged 21.4 to 51 with a mean age of 38). Study sample sizes ranged from 37 to 312 (mean = 147).	Individual Placement and Support (IPS)	21 RCT studies were included, published between 1996 and 2015.	Competitive employment rate
22	Modini et al., 2016	To investigate whether IPS is effective across international settings and in different economic conditions.	4504 subjects were part of the 19 studies, the ages were not reported. The study participants had SMIs.	Individual Placement and Support (IPS)	17 RCT studies and 2 follow-up studies were included, published between 1996 and 2015.	Rate of competitive employment
23	Moen et al., 2020	To explore comprehension of the experiences of individuals applying for employment, employment specialists, social workers in welfare services, and clinicians in mental healthcare services within the context of individual placement and support (IPS).	327 participants (197 clients, 117 employment specialists, 10 clinicians, and 3 social workers).	Individual Placement and Support (IPS)	17 qualitative studies using ethnography, case study, content analysis, grounded theory, thematic analysis, or phenomenological analysis. Studies were published between 2001 and 2017.	The complexities and relationships among the experiences of these groups and to uncover elements that might contribute to collaboration difficulties within the IPS framework.
24	Suijkerbuijk et al., 2017	To assess the comparative effectiveness of various types of vocational rehabilitation interventions and to rank these interventions according to their effectiveness to facilitate competitive employment in adults with severe mental illness.	Adults aged (18–70) with diagnosed severe mental illness. 8743 participants were recorded within 48 RCTs (Average of 182/study).	Prevocational training: Job-related skills training, Symptom-related skills training, Cognitive training, Social skills training; Transitional employment: Sheltered workshop, Social enterprise, Clubhouse model; Supported employment: Low-fidelity IPS/not IPS, High-fidelity IPS; Augmented supported employment: Supported employment + job-related skills training, Supported employment + symptom-related skills training, Supported employment + sheltered employment; Psychiatric care only: Assertive Community Treatment.	48 RCTs were included, published between 1963 and 2015.	Percentage/number of participants that obtained competitive employment, number of weeks in competitive employment, number of days to first competitive employment, percentage of participants who obtained non-competitive employment (such as employment in a sheltered workplace or volunteer work), quality of life (e.g. QOLI), mental health (psychiatric symptoms) (e.g. PANSS), adverse events (dropouts, hospital admissions).
25	Twamley et al., 2003	To summarise the results of the investigations that contribute to evidence-based practice in vocational rehabilitation for people with severe mental illness.	The total number of participants studied was 1,617, with a mean sample size of 147 (range: 56 to 439). The majority (66%) of participants had a primary psychotic disorder. Participants were generally young (weighted mean age = 38 years), and 58% were male.	1. IPS or supported employment 2. Job-related social skills training	11 RCT studies were included, published between 1986 and 2002.	Outcomes measures were related to competitive employment, weeks worked, hours worked, and wages earned.
				3. Incentive Therapy, a VA-based program that offers part-time, set-aside job placements at the VA hospital, compensated at rates below the national minimum wage.		
26	Wallstroem et al., 2021	To evaluate the correlations between Individual Placement and Support (IPS), job attainment, and personal as well as clinical recovery in individuals with severe mental illness at an 18-month follow-up.	Participants were unemployed adults of either sex or ages 18–65, with SMI. A total of 1056 participants were reported.	Individual Placement and Support (IPS)	6 RCTs for meta-analyses, and pooled original data from 5 studies. The studies were published between 1999 and 2019.	Outcome measures related to self-esteem, empowerment, quality of life, hope, self-efficacy, depression, psychotic and negative symptoms, anxiety, and level of functioning.

#### Employment outcomes (*n =* 23 review studies)

Employment outcomes refer to the quantifiable consequences individuals encounter within their efforts related to preparing for, seeking, obtaining, maintaining, and regaining employment. Twenty-three reviews employed vocational outcome indicators to assess the effectiveness of interventions. These indicators included aspects such as employment rates, time taken to secure initial employment, job tenure, total hours or days worked, and instances of job changes. Some reviews incorporated educational outcomes in conjunction with competitive employment outcomes, particularly when evaluating programs tailored for young individuals [42, 63].

*Standard Supported Employment*. Most included reviews focused on the IPS model, a competitive employment intervention, except for one conducted by McKay et al. [55] who assessed the Clubhouse model (albeit arguing a competitive element in the interview for membership). Supported employment exhibited diverse employment outcomes, collectively across outcome indicators: 60% (*n* = 18) reported positive effects, 30% (*n* = 9) showed mixed results, and 10% (*n =* 3) indicated no effective results, based on one or more indicator: seeking, obtaining, maintaining, and regaining employment (see Table 6, and also refer to Table 5 for intervention typologies against the main outcome metrics for each review study).

**Table 6:** Outcomes reported in association with support model.

Outcomes	Interventions	Effective	Mixed results	Ineffective
Employment	Supported Employment (SE)	60% (*n* = 18)	30% (*n* = 9)	10% (*n* = 3)
	Augmented Supported Employment (ASE)	71% (*n* = 12)	23% (*n* = 4)	6% (*n* = 1)
	Vocational Rehabilitation and Training (VRT)	30% (*n* = 6)	40% (*n* = 8)	30% (*n* = 6)
	Standard Care (SC)	0% (*n* = 0)	33% (*n* = 1)	67% (*n* = 2)
Quality of Life	Supported Employment (SE)	50% (*n* = 5)	0% (*n* = 0)	50% (*n* = 5)
	Augmented Supported Employment (ASE)	100% (*n* = 1)	0% (*n* = 0)	0% (*n* = 0)
	Vocational Rehabilitation and Training (VRT)	0% (*n* = 0)	0% (*n* = 0)	0% (*n* = 0)
	Standard Care (SC)	0% (*n* = 0)	0% (*n* = 0)	0% (*n* = 0)
Social Functioning	Supported Employment (SE)	29% (*n* = 2)	14% (*n* = 1)	57% (*n* = 4)
	Augmented Supported Employment (ASE)	100% (*n* = 1)	0% (*n* = 0)	0% (*n* = 0)
	Vocational Rehabilitation and Training (VRT)	0% (*n* = 0)	0% (*n* = 0)	0% (*n* = 0)
	Standard Care (SC)	0% (*n* = 0)	0% (*n* = 0)	0% (*n* = 0)
Clinical/Service Use	Supported Employment (SE)	30% (*n* = 3)	20% (*n* = 2)	50% (*n* = 5)
	Augmented Supported Employment (ASE)	67% (*n* = 2)	0% (*n* = 0)	33% (*n* = 1)
	Vocational Rehabilitation and Training (VRT)	56% (*n* = 5)	22% (*n* = 2)	22% (*n* = 2)
	Standard Care (SC)	0% (*n* = 0)	0% (*n* = 0)	0% (*n* = 0)
Economic	Supported Employment (SE)	22% (*n* = 2)	33% (*n* = 3)	45% (*n* = 4)
	Augmented Supported Employment (ASE)	78% (*n* = 7)	22% (*n* = 2)	0% (*n* = 0)
	Vocational Rehabilitation and Training (VRT)	40% (*n* = 2)	0% (*n* = 0)	60% (*n* = 3)
	Standard Care (SC)	0% (*n* = 0)	0% (*n* = 0)	0% (*n* = 0)

Note: ’*n*’ denotes the number of studies contributing to each percentage.

Diverse results underscored the need for context-specific evaluation of supported employment effectiveness. For instance, Abidin et al. [64], Bond et al. [40], Bond et al. [42], and Frederick and VanderWeele [65] consistently reported that IPS effectively promoted competitive employment rates, expedited job acquisition, and extended job tenure compared to control groups. Control groups were locally available practice which complicated the ability to generalise results within and across review studies. Campbell et al. [66] reported on results stratified by subgroups, such as participants with higher education levels or those who were divorced, showing that IPS did not exhibit a significant impact. While IPS had a positive impact on obtaining employment for young people experiencing psychosis, keeping a job was shown in another study to remain a challenge [63]. Carmona et al. [67] noted diverse findings which were due to various associations between IPS and job competitiveness, job placements and job tenure across primary studies included, ranging from IPS having no statistically significant effects to significant positive impacts. Dewa et al. [68] reviewed IPS primary studies compared Augmented Supported Employment with Standard Employment. They showed that augmented IPS achieved higher competitive employment rates than standard IPS. They stressed that all primary studies in their review had a moderate to high risk of bias; results should be viewed with caution.

Noting that IPS was originally developed in the US [26], several reviews examined and found reports of effectiveness of IPS across international settings [52, 53]. Bond et al. [69] found that IPS programs outperformed control groups, which exclusively comprised either standard treatment or established alternative vocational models like vocational rehabilitation, in achieving competitive employment outcomes and retention rates. Additionally, the success rates for IPS participants in US primary studies significantly surpassed those in non-US primary studies. Modini et al. [23] concluded that IPS was relatively effective, based on higher GDP growth. Metcalf et al. [54] made a compelling argument, highlighting the critical role of regulatory moderators as either enablers or impediments to the overall effectiveness of IPS. Mallick and Islam [52] investigated the effect of co-location partnerships between adult mental health and disability employment services in Australia. Their review showed that IPS was effective, however the implementation encountered hurdles related to awareness and training, complex guidelines, fidelity struggles, and undervaluation recovery principles. Twamley et al. [70] indicated that, although IPS appeared to be the most effective type of employment interventions, nearly half of IPS participants in primary studies reviewed did not obtain competitive work at any time. In the UK context, IPS primary studies showed mixed results in competitive employment rates [53]. Kinoshita et al. [71] also found mixed results on various employment outcomes in their review of clinical trials internationally, concluding that there was no evidence of IPS fidelity affecting job tenure for any paid employment. The Clubhouse model was effective in helping individuals with SMI obtain and maintain competitive employment [55]. However, Suijkerbuijk et al. [72] found that the model was no more effective than psychiatric care alone.

*Augmented supported employment*. Twelve reviews have highlighted the positive impact (71%) of augmented supported employment on employment outcomes; 23% (*n* = 4) were mixed and 6% (*n =* 1) of reviews reported limited impact (Table 6). Abidin et al. [64] reported higher employment rates, longer job tenures, and increased job success in IPS plus social skills training groups compared to control groups; controls typically being treatment as usual or vocational rehabilitation services. Aguey-Zinsou et al. [63] found that integrating IPS with unpaid internships for young people led to employment rates of at least 20 hours per week that were significantly higher over both one and two years compared to alternative vocational strategies. Dewa et al. [68] specifically reviewed Augmented Supported Employment and noted that adding cognitive training or specific skills programs to IPS improved employment outcomes and job stability, with Augmented Supported Employment consistently out-performing IPS and traditional vocational rehab. Johanson et al [56] and Suijkerbuijk et al. [72] reached a similar conclusion on cognitive training with IPS. Suijkerbuijk et al. [72] identified other effective combinations, such as IPS augmented with symptom-related skills or sheltered employment. However, caution should be exercised when considering complex combinations, as they may yield counterproductive results. An illustration of this is the combination of neurocognitive enhancement therapy with a hybrid model of transitional and IPS, which resulted in adverse job tenure outcomes [67]. Likewise, Abidin et al. [64] found that there was no statistically notable difference between cohorts receiving Augmented Supported Employment combined with cognitive remediation and cohorts undergoing Augmented Supported Employment without this additional element.

*Vocational rehabilitation and training*. Vocational rehabilitation, industrial therapy, prevocational training with payment incentives, prevocational training combined with psychosocial interventions, job-related social skills training, and incentive therapy, have consistently demonstrated positive impacts on employment outcomes [63, 70, 73]. Interventions such as combining behavioural strategies (work performance feedback and goal setting) with paid job placement, cognitive enhancement therapy, and vocational integrated programs involving Assertive Community Treatment and family psychoeducation groups have yielded mixed results [67]. Approximately 30% (*n* = 6) of the Vocational Rehabilitation and Training primary studies included in the reviews reported effective outcomes, while 40% (*n* = 8) indicated mixed results, and the remaining 30% (*n* = 6) suggested ineffective outcomes based on one or more indicator: seeking, obtaining, maintaining, and regaining employment (Table 6). While a substantial proportion of primary studies showed promise, the variations in outcomes suggest that the effectiveness of Vocational Rehabilitation and Training interventions may depend on contextual factors.

*Standard care*. There are limited reviews available that report on Standard Care interventions, with Standard Care often used as a control group. Limited reviews have discussed the effectiveness of standard care programs, which include Community Treatment Orders, Early Intervention, and Medication (including discontinuation). However, these interventions did not demonstrate a significant positive impact on employment related outcomes [63, 72].

**Fig 3:**
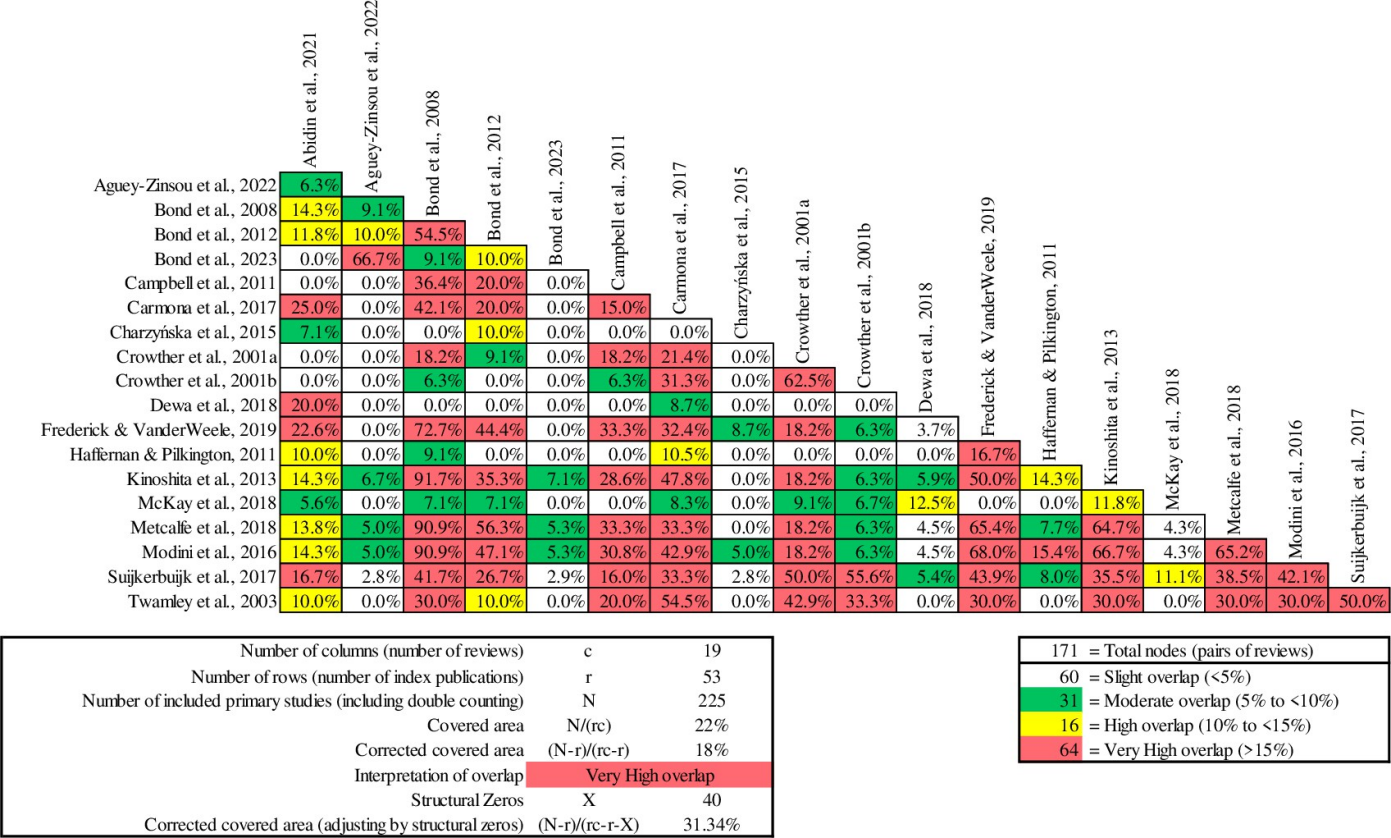
Pairwise CCA for reviews reporting employment outcomes.

An analysis of the 18 reviews assessing employment outcomes revealed significant overlap, exceeding 90% (Fig 3), particularly in the reviews by Bond et al. [40], Kinoshita et al. [71], Metcalf et al. [54], and Modini et al. [23], which covered 11, 14, 21, and 17 RCTs, respectively. All reviewers reached a consensus that, in general, IPS led to significantly improved competitive employment outcomes compared to control groups. However, the data exhibited substantial heterogeneity in which meta-regressions showed that neither country region, unemployment rate, or other variables, were responsible, demanding a careful and nuanced interpretation to avoid drawing misleading conclusions. While the reviews share a primary focus on examining the effectiveness of the IPS model, each review also presents unique additional focuses or contexts. For instance, one review might delve into IPS effectiveness in an international comparative context. Bond et al. [40] assessed IPS effectiveness in both U.S. and non-U.S. context, Kinoshita et al. [71] found mixed impacts on vocational and non-vocational outcomes, whereas Modini et al. [23] highlighted IPS effectiveness in different GDP settings, and Metcalfe et al. [54] emphasised the role of regulatory moderators in influencing IPS outcomes. This variation both within and between the reviews collectively provides a richer context for assessing the impact of IPS programs on employment outcomes.

#### Quality of life (*n =* 7 review studies)

Seven reviews focused on primary studies administering quality of life measures to explain intervention effect across life domains. The Standard Supported Employment approach (including IPS) yielded mixed results, with 50% of review studies (*n* = 5) reporting effective outcomes, while the remaining 50% (*n* = 5) did not report any notable impact (e.g., ineffective—Table 6). Subjective quality of life measures indicated that Augmented Supported Employment had beneficial outcomes for individuals across these review studies. There were no Vocational Rehabilitation Training or Standard Care reviews reporting on quality-of-life measures.

Abidin et al. [64] reported a significant positive effect of IPS on the quality of life of participants, particularly when there is a strong emphasis on occupational engagement within IPS programs. Frederick et al [65] also observed higher levels of quality of life among individuals in IPS conditions. While scales differed across primary studies, the Quality of Life Interview was most typical. In addition, Wallstroem et al.’s [61] observation that IPS participants were employed for longer durations and showed more improvements in quality of life, underscoring the importance of sustained employment. However, Crowther et al. [73, 74] found no significant difference in the quality of life between IPS and other Standard Supported Employment participants, regardless of the approach used. Kinoshita et al. [71] found no clear indications that Supported Employment or high fidelity IPS had a significant impact on average endpoint quality of life scores across different domains.

**Fig 4:**
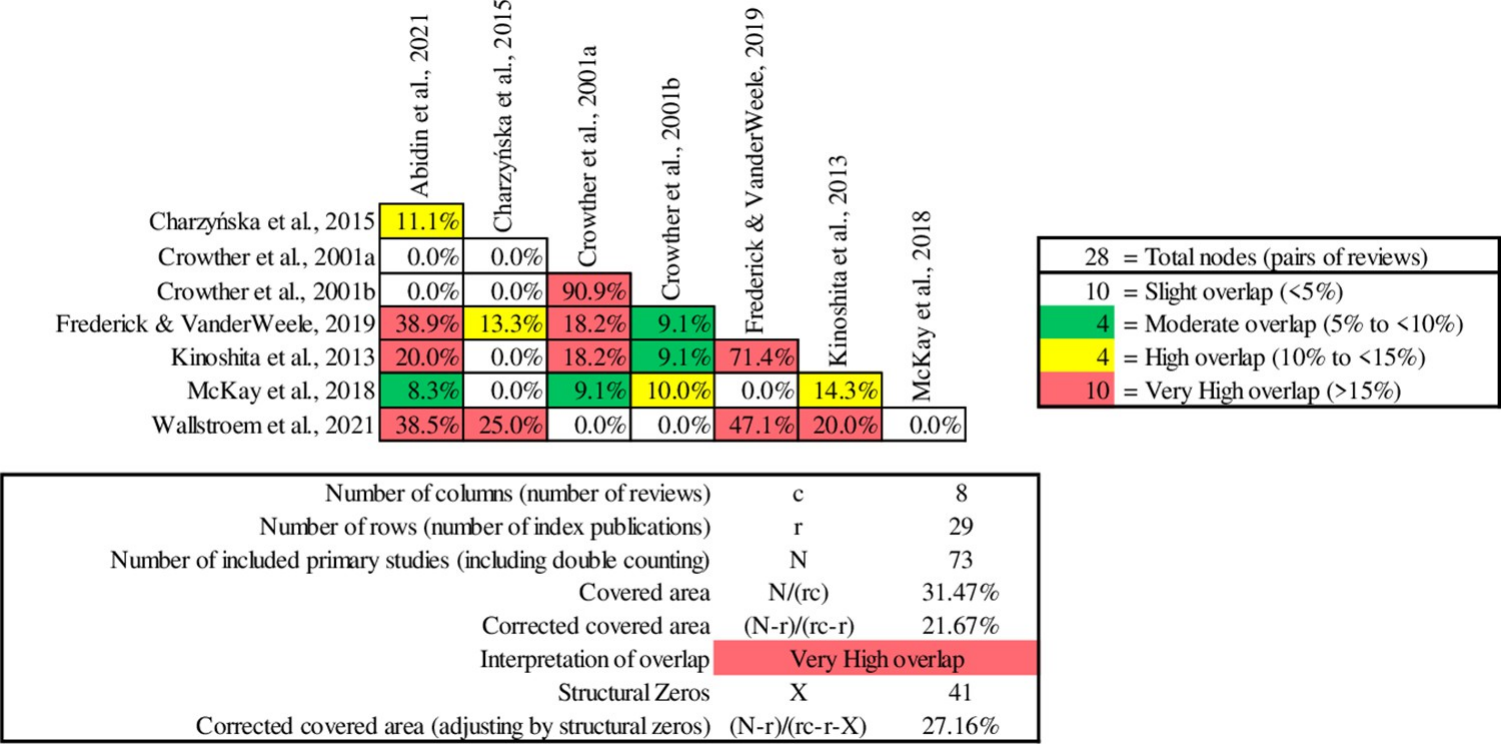
Pairwise CCA for reviews reporting quality of life outcomes.

The Clubhouse model, as highlighted by McKay et al. [55], demonstrated effectiveness in enhancing various aspects of quality of life. Augmented Supported Employment (IPS with social skills training) showed notable positive impacts on overall life outcomes [64]. These findings underscore the profound impact of meaningful work and sustained employment across quality of life domains, emphasising the importance of both securing and maintaining jobs over time. They suggest that the choice of vocational approach alone may not be the sole determinant for achieving a good life. Pairwise CCA (Fig 4) conducted on reviews that reported quality of life and showed a very high overlap, specifically between Crowther et al., [74] and [73] reviews, where the overlap exceeded 90%. Both reviews consistently concluded that neither IPS nor any approach of Standard Supported Employment had a significant impact on quality of life.

#### Social functioning (*n =* 5 review studies)

Within the domain of social functioning outcomes, Standard Supported Employment showed 29% (*n* = 2) effective outcomes, 14% (*n* = 1) mixed results, and 57% (*n* = 4) showed no effective outcomes (Table 6). While several reviews have discussed Augmented Supported Employment, specifically IPS with social skills training, only one review specifically measured outcomes related to social functioning, with positive results reported (Table 6). This review of Augmented Supported Employment [64] identified positive outcomes on social functioning. They proposed that integrating IPS with specific social skills training was vital for promoting better social interactions, communication, and overall social well-being for individuals engaging in vocational programs. There were no Vocational Rehabilitation and Training or Standard Care review studies reporting on social functioning.

**Fig 5:**
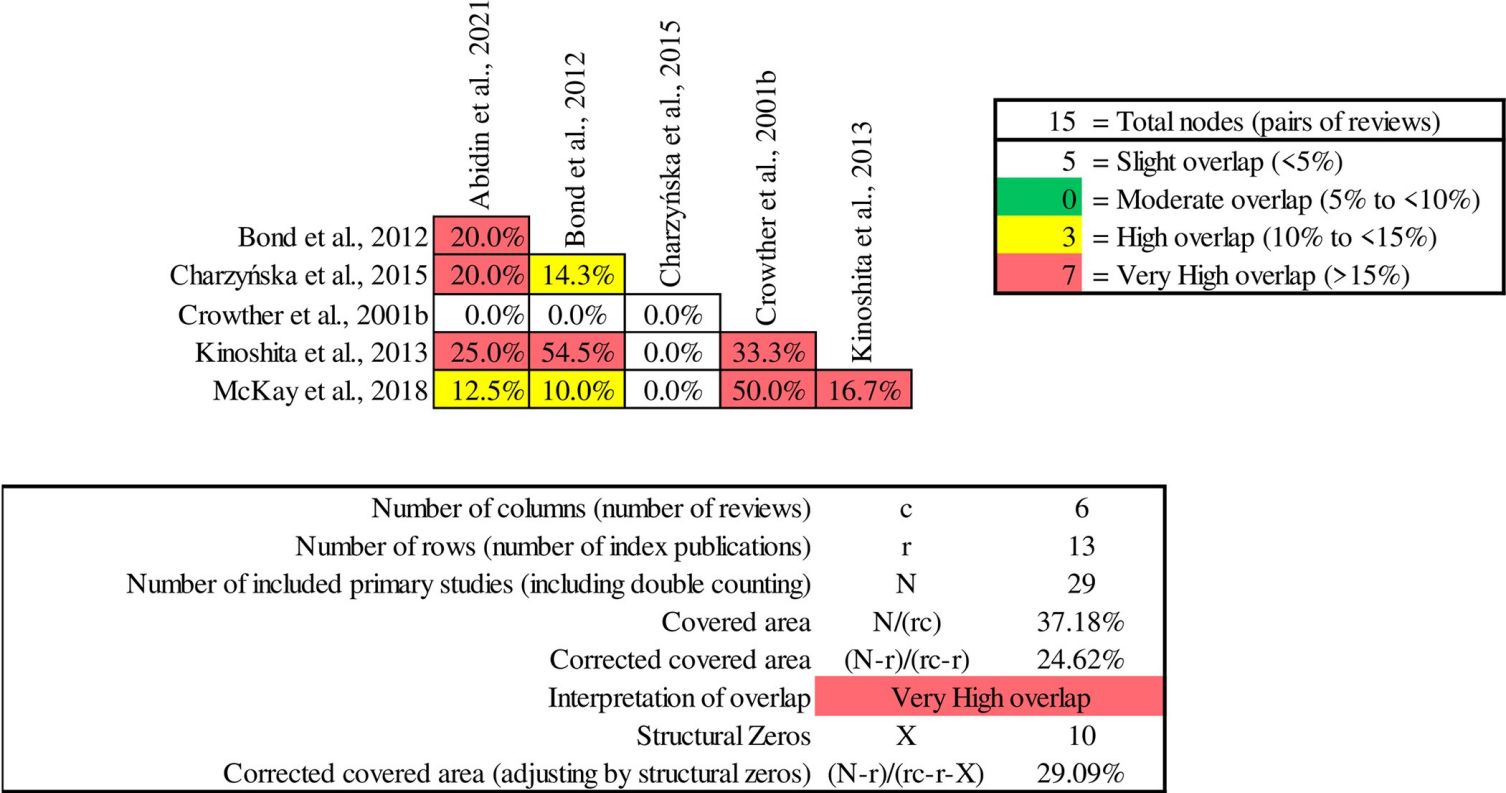
Pairwise CCA for reviews reporting social functioning outcomes.

Although social functioning outcomes were not significantly associated with IPS or other standard employment models, Abidin et al. [64] showed in some primary studies reductions to social disability (i.e., less physical, attitudinal, communication, and social barriers in the workplace). Bond et al. [69] did not observe significant differences in social functioning and network measures for IPS participants compared to controls. Crowther et al. [73] found no notable differences in social functioning between the groups studied. Similarly, Kinoshita et al. [71] highlighted no clear link between supported employment and either lower or higher social functioning scores compared to alternative approaches in the long run. Furthermore, the study emphasised that high fidelity IPS did not exhibit discernible advantages in this domain. A contrasting outcome was highlighted by McKay et al. [55] showcasing the effectiveness of the Clubhouse model in enhancing various social aspects for individuals grappling with severe mental health challenges. Abidin et al. [64] found positive evidence for improved social functioning and reduced interpersonal conflicts in the workplace for Augmented Supported Employment (IPS plus social skills training) participants. The inception of this approach primarily aimed to enhance IPS by addressing gaps in social and interpersonal functioning (Fig 5).

#### Clinical and service utilisation outcomes (*n =* 10 review studies)

*Standard supported employment*. In terms of clinical and service utilisation outcomes, 50% of Standard Supported Employment review studies demonstrated no difference results, followed by a smaller percentage of positive outcomes (30%), and an equal percentage of mixed results (20%) (Table 6). The available data regarding mental health or clinical and service utilisation outcomes in the context of supported employment interventions is notably limited in specificity. Within the review findings, several reviews [56, 69, 71, 73, 74] revealed no statistically significant disparities in clinical outcomes (e.g., mental functioning, psychiatric symptoms, hospitalisations). Conversely, Abidin et al. [64] review noted a decrease in psychiatric symptom occurrence, reduced service dropout rates, and decreased re-hospitalisation instances within IPS groups in comparison to control conditions. Analyses by Bond et al. [69] and Crowther et al. [73] disclosed that participants in IPS did not manifest discernible differences from control groups in psychiatric symptoms and psychiatric hospitalisations. Standard Supported Employment approach, as elucidated by Crowther et al. [74], showed no significant deviation in hospital admissions and symptom severity compared to Standard Care. Kinoshita et al. [71] underscored the absence of compelling evidence to support associations between high fidelity IPS with either reduced or increased symptom scores or hospitalisation rates when juxtaposed with alternative vocational methodologies.

Crowther et al. [73] reported no distinctions in the frequency of hospital admissions between Standard Supported Employment groups and those receiving Standard Care, extending this lack of discrepancy to overall functioning and mental state across all Standard Supported Employment approaches. Nonetheless, Wallstroem et al. [61] observed a reduction in negative symptoms among employed participants, constituting a clinically relevant finding, and discerned no adverse clinical implications associated with IPS participation. Frederick and VanderWeele [65] noted a potential positive impact of IPS on global functioning and mental health but acknowledged the possibility of no significant effect. An efficacious reduction in the number and duration of hospitalisations among individuals was documented within the Clubhouse model [55].

*Augmented supported employment*. Augmented Supported Employment model showed potential positive effect on psychiatric symptoms, with 67% of included reviews showing a positive overall result (Table 6). For example, Abidin et al. [64] showed from a few primary studies that the combination of IPS and social skills training resulted in positive psychological outcomes, indicated by higher scores on both the Brief Psychiatric Rating Scale (BPRS) and the Global Assessment of Functioning (GAF) compared to standalone IPS paired or Standard Vocational Rehabilitation. Likewise Augmented Supported Employment showed positive effect in cognitive functioning, as in the review by Johanson et al. [56] where several primary studies combined IPS with cognitive remediation groups resulting in significant improvements to cognitive functioning. In contrast, incorporating Assertive Community Treatment into a Standard Supported Employment program did not demonstrate a substantial difference in hospital admissions when compared to the Standard Care group, as found by Crowther et al. [74].

**Fig 6:**
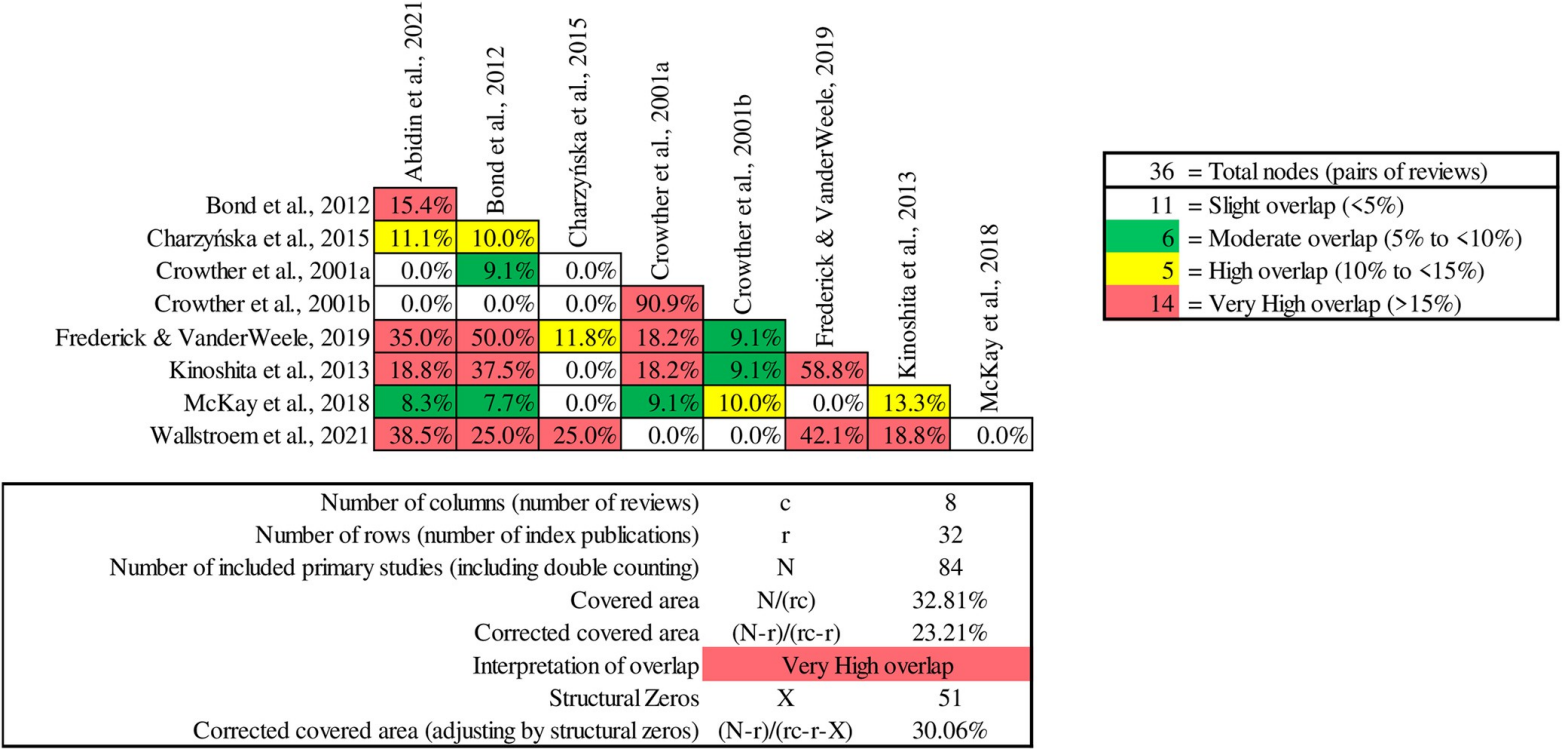
Pairwise CCA for reviews reporting clinical and service utilisation outcomes.

In terms of clinical and service utilisation outcomes, Augmented Supported Employment overall outperformed Standard Supported Employment. Vocational Rehabilitation and Training yielded even more positive results than Standard Supported Employment (Table 6). Vocational rehabilitation and pre-vocational training were associated with a decrease in hospital admissions for individuals with SMI [64, 73, 74]. These programs demonstrated a potential positive impact on hospitalisation rates. Pairwise CCA for reviews reporting clinical and service utilisation outcomes, a notable overlap of over 90% was observed in two reviews by the same authors [73, 74], consistently indicating that Standard Supported Employment was not superior to Vocational Rehabilitation and Training (Fig 6).

#### Economic outcomes (*n =* 10 review studies)

In this context, economic outcomes include wages or salaries earned by individuals with SMI, direct cost which pertains to expenses incurred for hospitalisation, medical treatments, and rehabilitation services, and indirect costs that are those generated by lost productivity and early retirement, and the economic benefits of the interventions. However, the main outcome metrics reported related to wages and individual health costs in which Augmented Supported Employment demonstrated superior performance compared to both Standard Supported Employment and Vocational Rehabilitation and Training, with reviews indicating respective positive results of 78%, 22%, and 0% respectively (Table 6). Some reviews discovered that there were no discernible differences in overall earnings between IPS participants and the control group [67, 69, 71]. Conversely, Crowther et al. [74] presented mixed results where program costs of Standard Supported Employment were greater than Standard Care, but healthcare costs were lower for Standard Supported Employment. Further to this, Crowther et al. [73] identified differing outcomes in trials, one trial showed significantly higher mean monthly earnings, while the other showed no difference. The IPS group did earn more from competitive employment, yet there was no significant difference in program costs or overall healthcare costs between IPS and pre-vocational training. In a recent study, Johanson et al. [56] reported a negative net benefit for IPS intervention, indicating higher costs associated with IPS than potential savings. The intervention incurred higher costs, and participants earned less compared to alternative approaches.

Augmented Supported Employment, which incorporates IPS along with work-related social skills training, was compared with IPS alone. The mean salary earned over a 7-month follow-up period was slightly lower for Augmented Supported Employment at 24.58 compared to IPS at 25.85. However, over an extended 11-month period, the Augmented Supported Employment groups surpassed IPS in earnings, with Augmented Supported Employment earning 25.56 compared to IPS at 20.99 [68]. This suggests that while Augmented Supported Employment initially had a slightly lower mean salary, it showed greater effectiveness in increasing earnings over the longer-term. The costs of mental health care were found to be lower in IPS; however, this was also the case for Augmented Supported Employment. The Augmented Supported Employment programs demonstrated cost-effectiveness through productivity gains and cost savings resulting from the reduced use of labour market services. Although there is no significant cost difference between Cognitive Remediation integrated in IPS and control groups, the probability curves for cost-effectiveness highlighted that even slight improvements, such as an increase in working days or enhanced cognitive abilities, substantially raised the likelihood (ranging between 70% and 95%) of achieving a cost-effective outcome [56]. In the case of the monthly total healthcare costs, individuals in the Vocational Rehabilitation and Training group had healthcare costs 56% higher than those receiving standard community care. Moreover, when comparing Vocational Rehabilitation and Training with standard hospital care, participants in the Vocational Rehabilitation and Training group were found to earn a higher income, approximately 81% more per month [73]. While Vocational Rehabilitation and Training may result in higher healthcare costs, it significantly boosts individuals’ earning potential, potentially indicating the overall cost-effectiveness and socioeconomic benefits of the Vocational Rehabilitation and Training approach.

**Fig 7:**
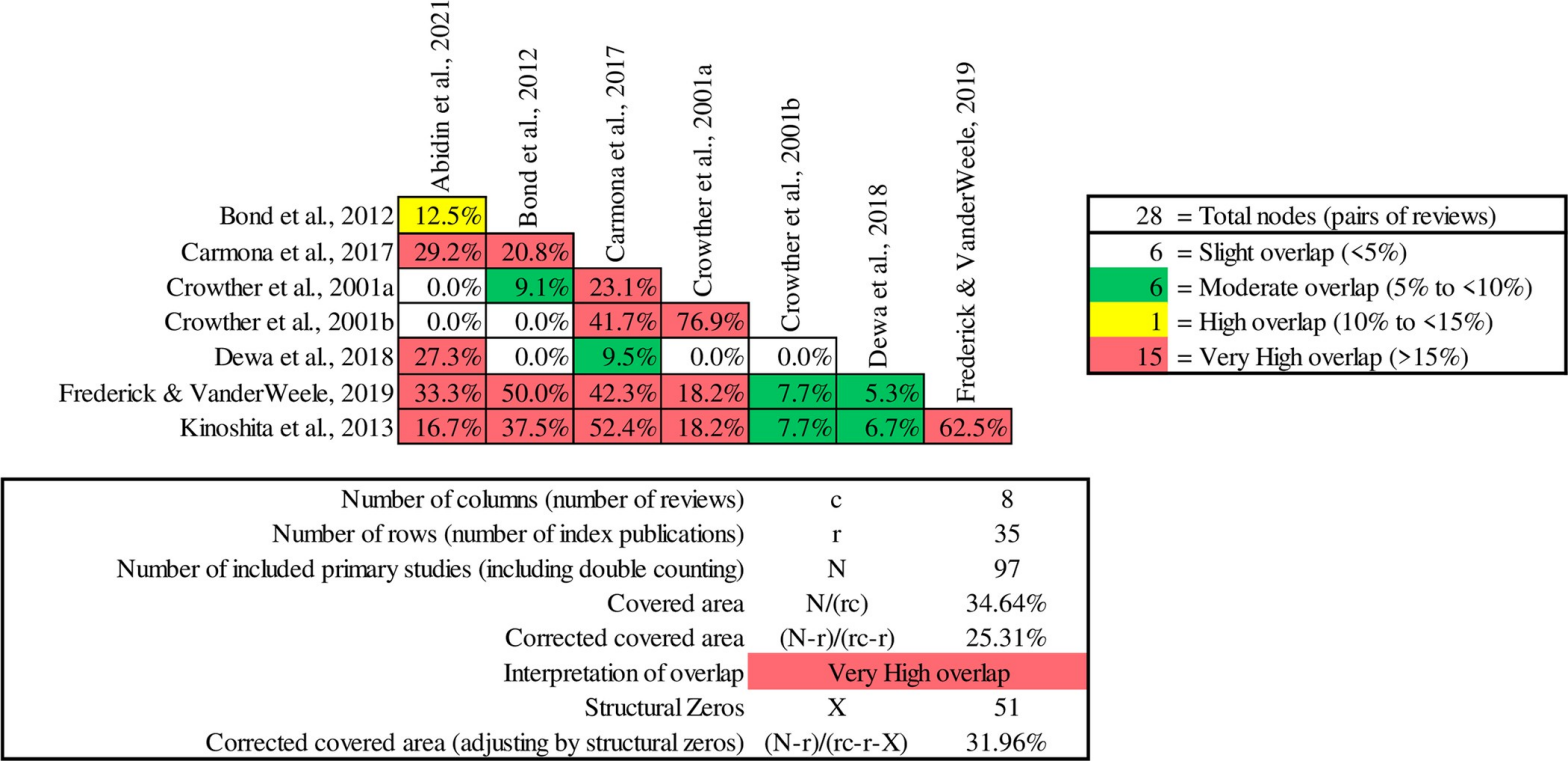
Pairwise CCA for reviews reporting economic outcomes.

An analysis of pairwise CCA for reviews reporting economic outcomes indicates a very high overlap of 31.96% across reviews. The highest overlap (76.9%) is between two reviews from the same authors (Fig 7). The insights provided by Crowther et al. [73] further substantiate findings from Crowther et al. [74], highlighting the notable cost disparities in healthcare and earnings between pre-vocational training and standard community care or standard hospital care in greater detail.

### Contextual factors influencing the outcome variability

**Fig 8:**
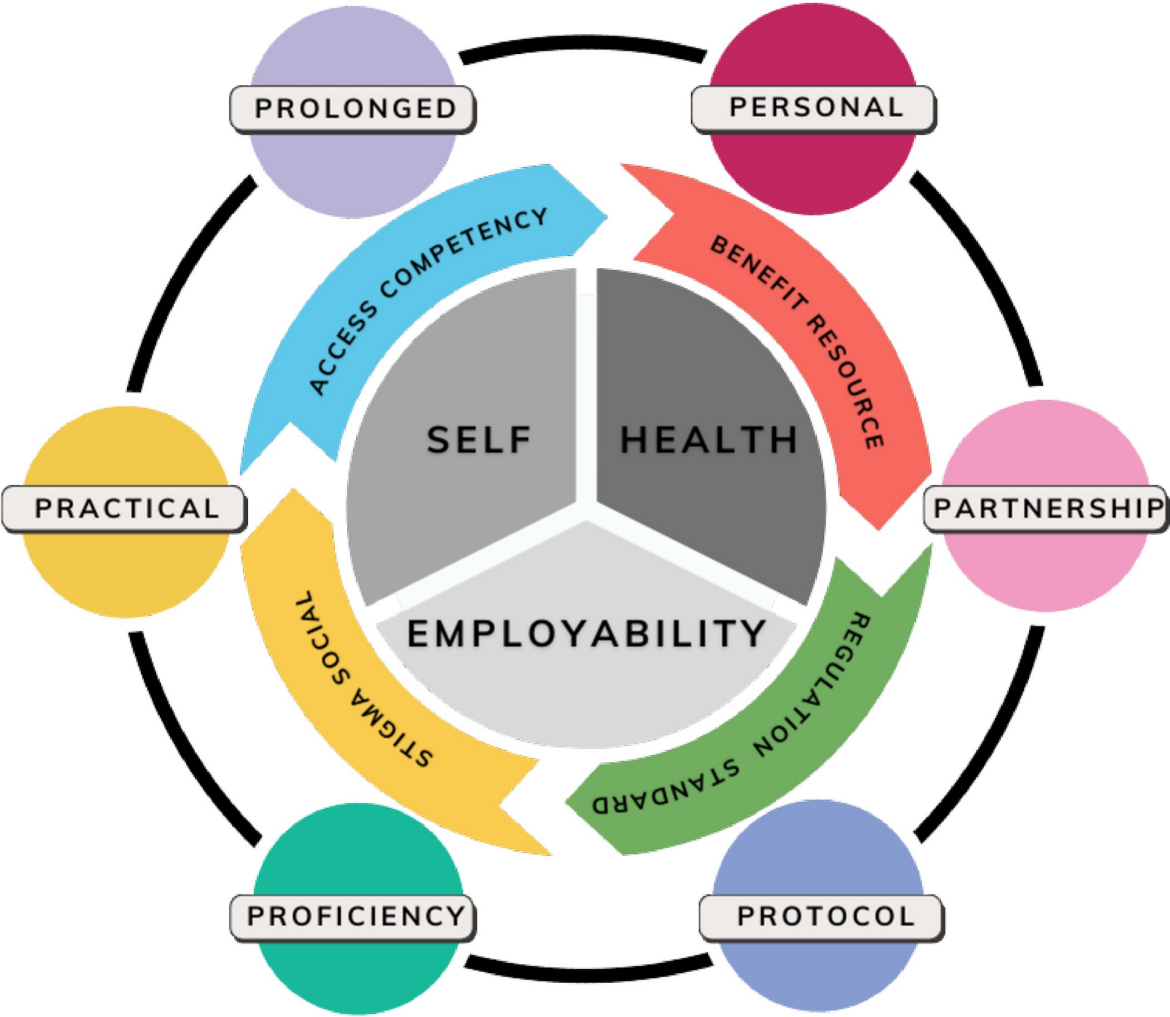
Factors influencing supported employment outcomes.

Out of the 26 reviews analysed, six revealed significant insights into the barriers that impede success and the vital supports essential for effective implementation in supported employment initiatives [52, 57–60, 63]. Qualitative insights from these review studies offer an understanding of the diverse factors contributing to outcome variability within this context. Fig 8 portrays a layered framework categorising factors influencing supported employment outcomes from these reviews, synthesised from collective themes identified in the six reviews [52, 57–60, 63]. The **inner circle** highlights **individual barriers** such as psychiatric *health* conditions; *self*-perception, fears, and motivation; and *work and skills* factors including work experience and skills. Charette-Dussault and Corbiere [57] found that psychiatric disabilities and their symptoms pose barriers to employment, but symptom severity alone does not consistently predict job acquisition. Positive symptoms such as hallucinations and delusions were not predictive, while certain negative symptoms such as social withdrawal or lack of motivation may have affected job acquisition. Depression, anxiety, and substance abuse show limited correlation with job acquisition. Cognitive deficits, medication side effects, and nonadherence have mixed relationships with employment. Specifically among young people, barriers to obtaining work identified were a more severe course of illness and treatment issues, such as hospital admission and medication side effects [63]. Personality psychopathological aspects and challenges in medication compliance are perceived as barriers to competitive employment [57]. Work and skills factors that include lack of work experience, underqualification, employment gaps, skill deficiencies, and unfamiliarity with job search strategies are strongest predictors of job acquisition. Social interaction difficulties and education levels are inconclusive to further impact job acquisition prospects [57]. Self-perception, poor self-confidence, and low self-esteem are perceived barriers to competitive employment, affecting motivation and job search efficacy. Lack of motivation is considered a major obstacle, but its direct relationship with job acquisition is inconsistent; expressing a desire for work does not always translate to active job search engagement. Many people with SMI may express fear and anxiety related to employment. This fear encompasses worries about their ability to cope with full-time work, handle job-related stress and pressure, and manage social interactions with employers and colleagues [57, 58].

The four elements in the middle circle (Fig 8) represents environmental barriers identified in four of the review studies, which encompass disability *benefits* and other physical *resources*, *intersecting stigma* and social support, *access* to vocational services and support provider *competencies*, and *regulation* and *standard* issues [57–59]. Fear of losing benefits led to reduced motivation and a passive job search [57, 58]. Challenges in physical resources such as transportation, childcare, and health insurance were barriers to employment for people with SMI [57]. Stigma surrounding severe mental illness creates a significant barrier to employment [57]. Disclosure concerns and negative perceptions from employers contribute to this issue [59]. Family and mental health service provider stigma, inadequate social support, and low expectations exacerbate the challenge. Individuals with SMI face challenges with coordination between vocational and broader rehabilitation services. Insufficient awareness of available employment services and a lack of person-centred support are major concerns. Job-seeking motivation and efforts are affected, with employment not always seen as a treatment priority by healthcare professionals [57]. Two review studies showed that government regulations posed challenges in navigating the job search process [52, 58]. Differing philosophies between IPS and government agencies affected job-seeking advice. Conflicting standards and expectations with external agencies caused challenges in collaboration. There was notable pressure on IPS to meet program targets, inadvertently hindering the smooth progression of the program [52, 58].

The outer circle (Fig 8) showcases various forms of support crucial for overcoming barriers and enhancing outcomes, including *personal* support, *practical* support, *prolonged* unlimited support, *partnership* and collaborative network support, *proficiencies* and attitudes, and *protocols* alignment that collectively contribute to a holistic supportive environment for individuals pursuing supported employment [58–60]. Personalised support is essential; people with SMI appreciate employment specialists’ encouragement, honesty, and flexibility. Acknowledging individual preferences and fostering a trusting relationship is foundational [60]. Employment symbolises hope, growth, and managing mental health impacts [59]. Practical support, including assistance with job search tasks such as resume writing and interviews are needed by individuals. The presence of an employment specialist during negotiations positively influenced perceptions, and employers highly appreciated employment specialist involvement in safety and problem-solving. Prolonged and unlimited support was vital for job search and maintenance. It provided individuals with a sense of security and helped during challenges. Partnerships and integrated support networks, where employment services are seamlessly embedded within mental health services, are highly valued by individuals. This co-located approach facilitates the development of realistic vocational plans that consider medical history, enhancing the collaboration between mental health and employment teams to effectively navigate system-level barriers [52]. Approval from the psychiatric team and flexible support workers are important. Moen et al. [59] implied that individuals highly valued the support of a network, friends, family, clinicians, and co-workers in achieving work goals. Moreover, essential proficiencies for employment specialists encompass teamwork, seamless integration of vocational assistance with mental health treatment, effective communication and translation skills, individual preparation for workplace expectations, employer education, symptom management support, and encouraging individual engagement in finding suitable job matches [59, 60]. Individuals stressed the employer’s crucial role in creating an inclusive work environment. Supportive employers, understanding and accommodating mental health challenges, were key to successful employment. Protocols alignment ensures harmonisation of recovery-oriented protocols among service providers and employers to support a unified vision, maximise efficiency, reduce conflicts, and enhance the overall effectiveness of the programs. Disclosures were underscored as pivotal elements affecting job retention. For example, Aguey-Zinsou et al. [63] emphasised the importance of a clear procedure regarding if and how individuals disclosed their health conditions to employers and work colleagues. Policies that did not prioritise inclusive employment could be identified as barriers in the reviews [52, 57, 58]. Advocating for policy changes that prioritise individual needs through person-centred protocols that align with welfare regulations is imperative.

The identified individual and environmental barriers give emphasis to the diverse challenges individuals with SMI which directly impacts the outcomes of efforts to attain and sustain employment. The supports mentioned, ranging from personalised support to policy synchronisation, aim to mitigate these barriers and enhance positive outcomes in sustainable employment.

## Discussion

This umbrella review considered extant evidence on supported employment models and their outcomes for people with severe mental illness, who experience some of the highest rates of unemployment and, when employed, poor working conditions and high rates of discrimination [1]. People with severe mental illness, in main, want to work and need support to sustain their workforce participation [e.g., 4–6]. However, factors such as disclosure of mental illness [75, 76], social disability [20], and other psychosocial aspects add to the challenges faced by individuals with SMI that deny their active citizenship [12, 13], as mentioned in some review studies.

Our review aligns with and expands upon the notion that employment is integral to recovery for people with SMI. Whitley and Drake [17] articulate that obtaining and maintaining valued societal roles and responsibilities, including employment, fosters a sense of purpose and belonging. Rights to employment, skill enhancement, interpersonal interactions, and engagement with community respects the roles and responsibilities of democratic societies to support social and productive opportunities for all. Consequently, Rowe and Davidson [14] argue that achieving active citizenship, for example employment, must not be delayed and take place as an integral component of treatment and recovery. In this comprehensive umbrella review, we consolidated existing review studies on supported employment with individuals with SMI, with a view to understanding optimal intervention responses.

In this review, we explored the effectiveness of supported employment interventions, specifically Standard Supported Employment (e.g., IPS) and Augmented Supported Employment, and their impacts on vocational and non-vocational outcomes. While Standard Care programs were not classified as supported employment interventions, they were included in our analysis to provide a comprehensive comparison and enhance understanding of the overall employment intervention landscape for individuals with SMI. It became evident in our synthesis of reviews that no singular approach is a panacea for supporting individuals with SMI. Rather, the efficacy of each intervention resides within its unique niche, contributing valuable threads to the broader fabric of mental health recovery. While Standard Supported Employment, particularly the IPS model, is demonstrated with superiority over alternative interventions, empirical evidence showed that its universal efficacy in sustaining employment is not guaranteed. Augmented Supported Employment, in contrast, exhibited superior performance in sustaining employment outcomes as well as improvements across remaining outcome indicators–quality of life, social functioning, clinical functioning and service use, and economic outcomes. Integrating supported employment with extended support systems and targeted interventions has proven effective in fortifying sustained job retention for individuals with SMI.

To complement these findings, our qualitative results provide contextual insight into the variability of outcomes. The synthesised framework from the six reviews consolidates comprehensive understanding of these factors, which range from individual barriers such as psychiatric health conditions, self-perception, and work-related skills to external influences such as social interaction difficulties and education levels. The variability in outcome determinants, for instance, exposes the mixed relationship between cognitive function and medication side effects with work capacity, underscoring the need for tailored approaches in supported employment programs. Managing psychiatric illness and symptoms is crucial, but recognising that symptom severity should not be the reason for denying job acquisition and employment support. The role of personal factors e.g., self-confidence, motivation, and fear of peer rejection in the workplace, can be moderated through tailoring person-centred supports. Augmented Supported Employment, with its tailored approach, effectively navigates the complexities of individual and external barriers which makes it a superior strategy for achieving sustainable employment for people with SMI.

### Strengths and limitations

Our umbrella review stands as a comprehensive synthesis of diverse review studies, providing a well-rounded perspective on the current landscape. It offers interdisciplinary insights valuable for informed decision-making. The reviews included a wide variety of study designs, from systematic reviews and meta-analyses to meta-ethnographic and scoping reviews. This methodological diversity, coupled with the broad range of participant characteristics and interventions across reviews, underscores the complexity of synthesising evidence in this field. Synthesis has been done so in so far as possible with a data set of heterogenous review studies with different focus and frameworks. The findings emphasise the comprehensive range of outcomes related to employment metrics, quality of life, social functioning, clinical, and economic outcomes, aligning with the holistic nature of recovery. Incorporating review studies of varying quality, from moderate to poor, was a deliberate choice to ensure a comprehensive overview of the evidence on supported employment interventions.

In addition to its strengths, the review is accompanied by key limitations. Variability in conclusions across review authors raises questions about overall reliability. The diverse range of primary studies introduces heterogeneity in study design and populations, potentially impacting generalisability and synthesis. The prominence of research on IPS interventions introduces publication bias in which volume of reporting could influence perceptions of effectiveness. It could be assumed that since IPS originated in the US, overrepresentation of US reviews might introduce bias, as could restricting the umbrella review to English-language publications. However, in this umbrella review, such bias could not be definitively assessed. Some review studies measured effectiveness outcomes, such as employment rates, time taken to secure initial employment, job tenure, total hours or days worked, and instances of job changes. The variability in measures in primary studies and across the included reviews implicated viability to undertake meta-analysis, or to examine potential differences between competitive and non-competitive employment outcomes. There were also time and resource constraints limiting ability to undertake further analysis.

## Conclusions

This umbrella review identifies gaps in existing knowledge, guiding future research endeavours, and fosters a direction for further exploration. Seven databases, four review study registers, and Google Scholar, were systematically search, identifying 26 review studies meeting the criteria. Specifically, our study indicates that focusing solely on vocational rehabilitation programs aimed at employment outcomes is insufficient for ensuring job stability, considering the substantial impact of illnesses on various aspects of life. A comprehensive strategy is essential, involving a dynamic understanding of factors such as unemployment rates, individual traits, vocational rehabilitation results, and a collaborative approach among healthcare, employment, and social services. In line with previous research, augmenting any form of supported employment and tailoring it to individual specific needs could be more successful than standardised models limited in person-centredness and individual relevance. The combination of supported employment with interventions targeting key challenges linked to mental illnesses, such as neurocognitive therapy and job-related social skill training, emerges as a promising approach to effectively address the complex causes of work-related challenges. Nonetheless, this umbrella review found that augmented supported employment has positive impact on employment and non-employment outcomes (e.g., health, quality of life, social functioning, wages) relative to standard forms of supported employment for people with SMI. Augmented supported employment interventions have the potential to be more widely applicable and adaptable to a broader range of employment outcomes for individuals with severe mental illness.

### Practice implications

Our umbrella review affirms that for individuals with SMI, employment outcomes are best served through a flexible, client-centred approach that transcends rigid model fidelity. Employment specialists are crucial for providing tailored support that is responsive to individual circumstances, contributing to enhanced job acquisition and retention. Integrating employment with mental health services through co-location and fostering collaboration with employers to develop inclusive workplaces is essential. This involves providing employer education on mental health, crafting accommodating work policies, and encouraging open dialogue for stigma-free communication. A dynamic, multidisciplinary strategy that emphasises personalised support and workplace adaptability aligns with the holistic recovery goals of individuals with SMI, promoting sustained meaningful employment and life satisfaction.

### Implications for future research

The identified overlaps among primary studies in our umbrella review highlight a significant methodological concern within the field. As the Individual Placement and Support is one of the most researched supported employment interventions, there is a significant accumulation of data that could potentially lead to double-counting and skew findings in meta-analyses. This may overshadow other emerging or less conventional employment support models that could be equally effective or more suited to certain populations or contexts. To mitigate these issues, we suggest the development of centralised registries or databases to track primary studies included in reviews, thereby averting redundancy.

## Supporting information

S1 Checklist PRIOR checklist for overviews of reviews. (PDF)

S2 Checklist PRISMA 2020 checklist. Note: The PRISMA checklist is required by PLOS, however we recognise the PRIOR checklist as the current preferred instrument for overviews of reviews (see S1 Checklist).
(PDF)

S1 File Search strategy. (PDF)
