# Supplementary material for: Supported employment interventions with people who have severe mental illness: Systematic mixed-methods umbrella review
Source: PLoS One. 2024 Jun 5;19(6):e0304527. doi: 10.1371/journal.pone.0304527 (PMC11152269; doi:10.1371/journal.pone.0304527)
Supplement: S1 File — (PDF) [file pone.0304527.s003.pdf]

S1 File. Search Strategy

Keyword search: ("severe mental illness" OR "serious mental illness" OR smi OR schizophreni* OR psychotic OR psychos?s OR ptsd OR post-traumatic OR mdd OR "major depressi*" OR bipolar OR schizoaffective OR "eating disorders" OR schizoid OR "personality disorder" OR ocd OR "obsessive compulsive") AND (employment OR job OR vocation* OR career) AND (support OR intervention OR assistance OR service OR "individual placement and support" OR "supported employment") AND ("systematic review" OR "scoping review" OR "mixed methods review" OR "mixed method review" OR "review study" OR "narrative review" OR "realist review" OR meta-analysis OR meta-synthesis OR "qualitative synthesis" OR "rapid review" OR "critically appraised topic").

CINAHL search history – 156 records

#	Query	Search Options	Results
S1	"severe mental illness" OR "serious mental illness" OR smi OR schizophreni* OR psychotic OR psychos?s OR ptsd OR post-traumatic OR mdd OR "major depressi*" OR bipolar OR schizoaffective OR "eating disorders" OR schizoid OR "personality disorder" OR ocd OR "obsessive compulsive"	**Expanders** - Apply equivalent subjects **Search modes** - Boolean/Phrase	155,851
S2	employment OR job OR vocation* OR career	**Expanders** - Apply equivalent subjects **Search modes** - Boolean/Phrase	222,263
S3	support OR intervention OR assistance OR service OR "individual placement and support" OR ips OR "supported employment" OR rehabilitation	**Expanders** - Apply equivalent subjects **Search modes** - Boolean/Phrase	1,681,008
S4	"systematic review" OR "scoping review" OR "mixed methods review" OR "mixed method review" OR "review study" "narrative review" OR "realist review" OR meta-analysis OR meta-synthesis OR "qualitative synthesis" OR "rapid review" OR "critically appraised topic"	**Expanders** - Apply equivalent subjects **Search modes** - Boolean/Phrase	234,456
S5	S1 AND S2 AND S3 AND S4	**Expanders** - Apply equivalent subjects **Search modes** - Boolean/Phrase	156

Scopus - 280 records

ALL ("severe mental illness" OR "serious mental illness" OR smi OR schizophreni* OR psychotic OR psychos?s OR ptsd OR post-traumatic OR mdd OR "major depressi*" OR bipolar OR schizoaffective OR "eating disorders" OR schizoid OR "personality disorder" OR ocd OR "obsessive

compulsive") AND TITLE-ABS-KEY ((employment OR job OR vocation* OR career) AND (support OR intervention OR assistance OR service OR "individual placement and support" OR ips OR "supported employment") AND ("systematic review" OR "scoping review" OR "mixed methods review" OR "mixed method review" OR "review study" "narrative review" OR "realist review" OR meta-analysis OR meta-synthesis OR "qualitative synthesis" OR "rapid review" OR "critically appraised topic"))

PubMed - 156 records

(("severe mental illness" OR "serious mental illness" OR smi OR schizophreni* OR psychotic OR psychos?s OR ptsd OR post-traumatic OR mdd OR "major depressi*" OR bipolar OR schizoaffective OR "eating disorders" OR schizoid OR "personality disorder" OR ocd OR "obsessive compulsive") AND (employment OR job OR vocation* OR career) AND (support OR intervention OR assistance OR service OR "individual placement and support" OR ips OR "supported employment")) AND ("systematic review" OR "scoping review" OR "mixed methods review" OR "mixed method review" OR "review study" "narrative review" OR "realist review" OR meta-analysis OR meta-synthesis OR "qualitative synthesis" OR "rapid review" OR "critically appraised topic")

Web of Science - 552 records

("severe mental illness" OR "serious mental illness" OR smi OR schizophrenia OR schizophrenic OR psychotic OR psychosis OR ptsd OR post-traumatic OR mdd OR "major depression" OR "major depressive" OR bipolar OR schizoaffective OR "eating disorders" OR schizoid OR "personality disorder" OR ocd OR "obsessive compulsive") AND (employment OR job OR vocation OR vocational OR career) AND (support OR intervention OR assistance OR service OR "individual placement and support" OR ips OR "supported employment") AND ("systematic review" OR "scoping review" OR "mixed methods review" OR "mixed method review" OR "review study" "narrative review" OR "realist review" OR meta-analysis OR meta-synthesis OR "qualitative synthesis" OR "rapid review" OR "critically appraised topic")

PsycINFO = 110 records

No.	Searches	Results
1	("severe mental illness" or "serious mental illness" or smi or schizophrenia or schizophrenic or psychotic or psychosis or ptsd or post-traumatic or mdd or "major depression" or "major depressive" or bipolar or schizoaffective or "eating disorders" or schizoid or "personality disorder" or ocd or "obsessive compulsive").mp. [mp=title, abstract, heading word, table of contents, key concepts, original title, tests & measures, mesh word]	506334
2	(employment or job or vocation or vocational or career).mp. [mp=title, abstract, heading word, table of contents, key concepts, original title, tests & measures, mesh word]	258419
3	(support or intervention or assistance or service or "individual placement and support" or ips or "supported employment").mp. [mp=title, abstract, heading word, table	1018997

	of contents, key concepts, original title, tests & measures, mesh word]	
4	("systematic review" or "scoping review" or "mixed methods review" or "mixed method review" or "review study narrative review" or "realist review" or meta-analysis or meta-synthesis or "qualitative synthesis" or "rapid review" or "critically appraised topic").mp. [mp=title, abstract, heading word, table of contents, key concepts, original title, tests & measures, mesh word]	77937
5	1 and 2 and 3 and 4	110

ProQuest - 108 records

abstract("severe mental illness" OR "serious mental illness" OR smi OR schizophrenia OR schizophrenic OR psychotic OR psychosis OR ptsd OR post-traumatic OR mdd OR "major depression" OR "major depressive" OR bipolar OR schizoaffective OR "eating disorders" OR schizoid OR "personality disorder" OR ocd OR "obsessive compulsive") AND abstract(employment OR job OR vocation OR vocational OR career) AND abstract(support OR intervention OR assistance OR service OR "individual placement and support" OR ips OR "supported employment") AND abstract("systematic review" OR "scoping review" OR "mixed methods review" OR "mixed method review" OR "review study narrative review" OR "realist review" OR meta-analysis OR meta-synthesis OR "qualitative synthesis" OR "rapid review" OR "critically appraised topic")

Emcare – 111 records

No.	Searches	Results
1	("severe mental illness" or "serious mental illness" or smi or schizophrenia or schizophrenic or psychotic or psychosis or ptsd or post-traumatic or mdd or "major depression" or "major depressive" or bipolar or schizoaffective or "eating disorders" or schizoid or "personality disorder" or ocd or "obsessive compulsive").mp. [mp=title, abstract, heading word, drug trade name, original title, device manufacturer, drug manufacturer, device trade name, keyword heading word]	175110
2	(employment or job or vocation or vocational or career).mp. [mp=title, abstract, heading word, drug trade name, original title, device manufacturer, drug manufacturer, device trade name, keyword heading word]	141797
3	(support or intervention or assistance or service or "individual placement and support" or ips or "supported employment").mp. [mp=title, abstract, heading word, drug trade name, original title, device manufacturer, drug manufacturer, device trade name, keyword heading word]	1244083
4	("systematic review" or "scoping review" or "mixed methods review" or "mixed method review" or "review study narrative review" or "realist review" or meta-analysis or meta-synthesis or "qualitative synthesis" or "rapid review" or	245557

	"critically appraised topic").mp. [mp=title, abstract, heading word, drug trade name, original title, device manufacturer, drug manufacturer, device trade name, keyword heading word]	
5	1 and 2 and 3 and 4	111

JBI EBP Database – 105 records

No.	Searches	Results
1	("severe mental illness" or "serious mental illness" or smi or schizophrenia or schizophrenic or psychotic or psychosis or ptsd or post-traumatic or mdd or "major depression" or "major depressive" or bipolar or schizoaffective or "eating disorders" or schizoid or "personality disorder" or ocd or "obsessive compulsive").mp. [mp=text, heading word, subject area node word, title]	643
2	(employment or job or vocation or vocational or career).mp. [mp=text, heading word, subject area node word, title]	632
3	(support or intervention or assistance or service or "individual placement and support" or ips or "supported employment").mp. [mp=text, heading word, subject area node word, title]	5321
4	("systematic review" or "scoping review" or "mixed methods review" or "mixed method review" or "review study narrative review" or "realist review" or meta-analysis or meta-synthesis or "qualitative synthesis" or "rapid review" or "critically appraised topic").mp. [mp=text, heading word, subject area node word, title]	7674
5	1 and 2 and 3 and 4	105

Campbell register – 0 records

Reviews matching "severe mental illness" or "serious mental illness" or smi or schizophrenia or schizophrenic or psychotic or psychosis or ptsd or post-traumatic or mdd or "major depression" or "major depressive" or bipolar or schizoaffective or "eating disorders" or schizoid or "personality disorder" or ocd or "obsessive compulsive" in Title Abstract Keyword AND employment or job or vocation or vocational or career in Title Abstract Keyword AND support or intervention or assistance or service or "individual placement and support" or ips or "supported employment" in Title Abstract Keyword AND "systematic review" or "scoping review" or "mixed methods review" or "mixed method review" or "review study narrative review" or "realist review" or meta-analysis or meta-synthesis or "qualitative synthesis" or "rapid review" or "critically appraised topic" in Title Abstract Keyword - (Word variations have been searched)

Cochrane Library – 18 records

Reviews matching "severe mental illness" or "serious mental illness" or smi or schizophrenia or schizophrenic or psychotic or psychosis or ptsd or post-traumatic or mdd or "major depression" or "major depressive" or bipolar or schizoaffective or "eating disorders" or

schizoid or "personality disorder" or ocd or "obsessive compulsive" in Title Abstract Keyword AND employment or job or vocation or vocational or career in Title Abstract Keyword AND support or intervention or assistance or service or "individual placement and support" or ips or "supported employment" in Title Abstract Keyword AND "systematic review" or "scoping review" or "mixed methods review" or "mixed method review" or "review study narrative review" or "realist review" or meta-analysis or meta-synthesis or "qualitative synthesis" or "rapid review" or "critically appraised topic" in Title Abstract Keyword - (Word variations have been searched)

Prospero – 0 records

Reviews matching "severe mental illness" or "serious mental illness" or smi or schizophrenia or schizophrenic or psychotic or psychosis or ptsd or post-traumatic or mdd or "major depression" or "major depressive" or bipolar or schizoaffective or "eating disorders" or schizoid or "personality disorder" or ocd or "obsessive compulsive" in Title Abstract Keyword AND employment or job or vocation or vocational or career in Title Abstract Keyword AND support or intervention or assistance or service or "individual placement and support" or ips or "supported employment" in Title Abstract Keyword AND "systematic review" or "scoping review" or "mixed methods review" or "mixed method review" or "review study narrative review" or "realist review" or meta-analysis or meta-synthesis or "qualitative synthesis" or "rapid review" or "critically appraised topic" in Title Abstract Keyword - (Word variations have been searched)
